# Vibrio cholerae Invasion Dynamics of the Chironomid Host Are Strongly Influenced by Aquatic Cell Density and Can Vary by Strain

**DOI:** 10.1128/spectrum.02652-22

**Published:** 2023-04-19

**Authors:** Dianshu Zhao, Afsar Ali, Cameron Zuck, Laurice Uy, J. Glenn Morris, Adam Chun-Nin Wong

**Affiliations:** a Entomology and Nematology Department, University of Florida, Gainesville, Florida, USA; b Genetics Institute, University of Florida, Gainesville, Florida, USA; c Emerging Pathogens Institute, University of Florida, Gainesville, Florida, USA; d Department of Environmental and Global Health, College of Public Health and Health Professions, University of Florida, Gainesville, Florida, USA; McGill University

**Keywords:** *Vibrio cholerae*, host-microbe interactions, microbiome, chironomids, microcosm

## Abstract

Cholera has been a human scourge since the early 1800s and remains a global public health challenge, caused by the toxigenic strains of the bacterium Vibrio cholerae. In its aquatic reservoirs, V. cholerae has been shown to live in association with various arthropod hosts, including the chironomids, a diverse insect family commonly found in wet and semiwet habitats. The association between V. cholerae and chironomids may shield the bacterium from environmental stressors and amplify its dissemination. However, the interaction dynamics between V. cholerae and chironomids remain largely unknown.  In this study, we developed freshwater microcosms with chironomid larvae to test the effects of cell density and strain on V. cholerae-chironomid interactions. Our results show that chironomid larvae can be exposed to V. cholerae up to a high inoculation dose (10^9^ cells/mL) without observable detrimental effects. Meanwhile, interstrain variability in host invasion, including prevalence, bacterial load, and effects on host survival, was highly cell density-dependent. Microbiome analysis of the chironomid samples by 16S rRNA gene amplicon sequencing revealed a general effect of V. cholerae exposure on microbiome species evenness. Taken together, our results provide novel insights into V. cholerae invasion dynamics of the chironomid larvae with respect to various doses and strains. The findings suggest that aquatic cell density is a crucial driver of V. cholerae invasion success in chironomid larvae and pave the way for future work examining the effects of a broader dose range and environmental variables (e.g., temperature) on V. cholerae-chironomid interactions.

**IMPORTANCE** Vibrio cholerae is the causative agent of cholera, a significant diarrheal disease affecting millions of people worldwide. Increasing evidence suggests that the environmental facets of the V. cholerae life cycle involve symbiotic associations with aquatic arthropods, which may facilitate its environmental persistence and dissemination. However, the dynamics of interactions between V. cholerae and aquatic arthropods remain unexplored. This study capitalized on using freshwater microcosms with chironomid larvae to investigate the effects of bacterial cell density and strain on V. cholerae-chironomid interactions. Our results suggest that aquatic cell density is the primary determinant of V. cholerae invasion success in chironomid larvae, while interstrain variability in invasion outcomes can be observed under specific cell density conditions. We also determined that V. cholerae exposure generally reduces species evenness of the chironomid-associated microbiome. Collectively, these findings provide novel insights into V. cholerae-arthropod interactions using a newly developed experimental host system.

## INTRODUCTION

A driving force for the emergence and reemergence of infectious diseases is the pathogen’s ability to adapt to changing environments, which includes the transitions between host and nonhost environments and across different host species. These adaptations can be reflected as phenotypic diversification of the pathogen governed by genetic changes and are influenced by a multitude of environmental factors. An example is the well-known human pathogen Vibrio cholerae, a globally distributed aquatic bacterium that causes endemic cholera in over 55 countries, with an estimated 1.3 to 4 million cases annually (1). The bacterium can inhabit a range of aquatic habitats and conditions but is also capable of spillovers from environmental reservoirs to infect humans (2–6). Despite the ongoing devastation by cholera, exactly how V. cholerae persists in the environment and emerges into outbreak strains is not well understood. A prevailing hypothesis is that active association with aquatic arthropods promotes the long-term survival of V. cholerae in aquatic ecosystems (7–9). For instance, the bacterium can derive nutrients from different biotic surfaces, such as chitins of exoskeletons (10, 11). Association with environmental hosts can also promote V. cholerae resistance to environmental stressors and may facilitate its evolution (12–14).

At present, there is a lack of empirical studies and tractable experimental host systems that explicitly consider V. cholerae environmental persistence with respect to its interactions with natural aquatic arthropod hosts. This type of assessment is immensely important in mapping the complex cholera transmission pathways toward epidemics and endemic scales, especially given the recent finding that aquatic reservoirs represent a critical source of toxigenic V. cholerae for recurrent outbreaks (15). In aquatic habitats, V. cholerae has been shown to be associated with arthropod hosts, including small crustaceans, chironomids (the nonbiting midges), and shellfish (16–18). Notably, V. cholerae can be detected at different life stages (eggs, larvae, pupae, and adults) of chironomids, both in the laboratory and in the wild (19–21). Chironomids are widespread arthropods in aquatic ecosystems, serving as primary producers and also a food source for higher animals, such as fishes and crustaceans. Chironomid larvae are also highly resilient to fluctuating water conditions, including tolerance to extreme drought, and interestingly, drought is associated with an increased prevalence of cholera outbreaks (22, 23). Taken together, the current evidence suggests that chironomids may be an important environmental host for V. cholerae. Investigating the extent to which different V. cholerae strains interact with the chironomids and the environmental contexts underlying V. cholerae-chironomid association will improve our understanding of how V. cholerae adapts to symbioses with aquatic hosts in order to optimize its environmental persistence and dissemination.

In this study, we leveraged chironomid larvae (Chironomus columbiensis) as a novel experimental host system, combined with laboratory freshwater microcosms, to determine the effects of bacterial cell density and strain on V. cholerae-chironomid interactions. By exposing the chironomid larvae to six different clinical isolates of toxigenic V. cholerae at four different inoculation doses, we found that V. cholerae exposure was generally nonpathogenic to the chironomid larvae unless reaching a critically high cell density. V. cholerae prevalence and load in the chironomid larvae generally correlated with inoculation dose, while interstrain variation in persistence and effects on host survival could be observed under specific cell density conditions. Microbiome analysis of the chironomid samples suggested that V. cholerae exposure can distort microbiome species evenness and the effect may be more general than strain specific. Together, our findings provide novel insights into V. cholerae invasion dynamics in chironomid larvae with respect to varying the aquatic cell density and strain.

## RESULTS

### Exposure to V. cholerae does not affect chironomid survival until reaching a critically high cell density.

**FIG 1:**
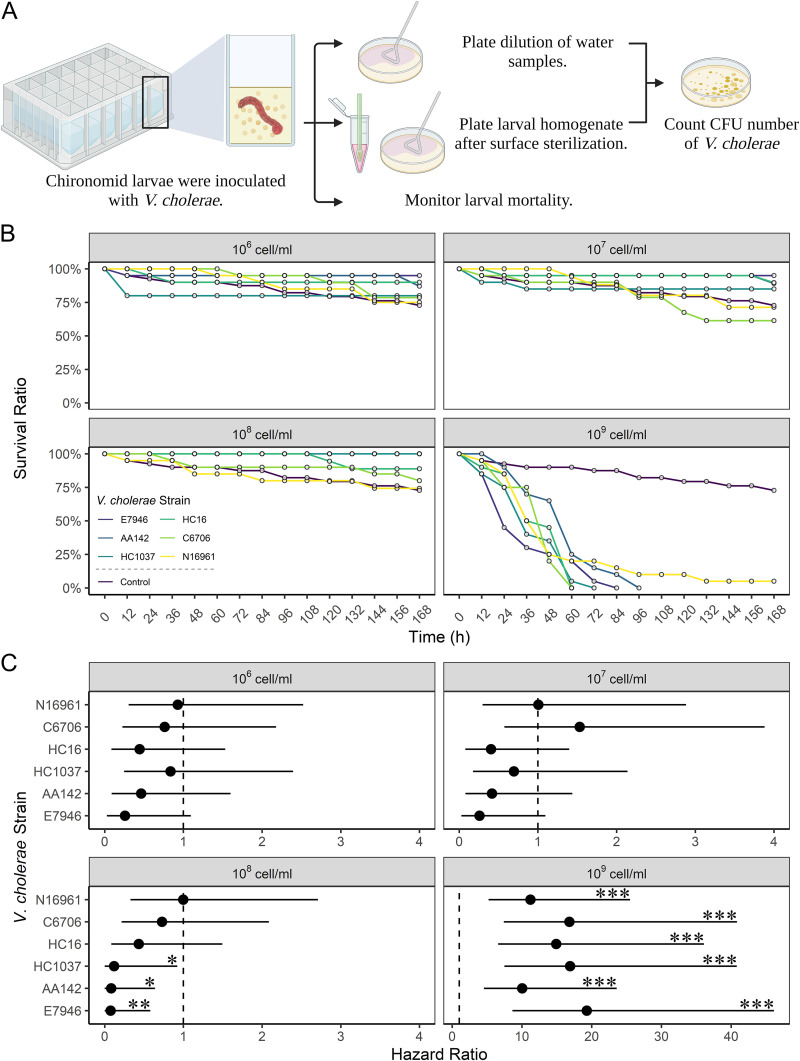
Survival ratio of chironomid larvae exposed to different V. cholerae strains at different inoculation doses. (A) Illustration of the experiments using microcosms. The chironomid larvae were exposed to V. cholerae in freshwater microcosms at four different cell densities (10^6^, 10^7^, 10^8^, and 10^9^ cells/mL). (B) Fractional survival was monitored and plotted every 12 h for 7 days (*n* = 20 microcosm wells of single larva per treatment). (C) Hazard ratio (HR) of larvae exposed to individual V. cholerae strains against the control (unexposed larvae). The error bars indicate 95% confidence intervals. HR values were calculated using Cox PH model with Firth’s penalized likelihood, which estimates the effect of treatment relative to the baseline (control). HR ranges from 0 to infinity. An HR of 1 indicates no effect, while an HR of >1 indicates a higher risk of death compared to the control. An HR of <1 means a lower risk, which suggests a survival benefit compared to the control. *, *P* < 0.05; **, *P* < 0.01; ***, *P* < 0.001.

To investigate if different V. cholerae strains can invade chironomid larvae in freshwater and the host response to various inoculation doses, 4th-instar chironomid larvae (12 to 14 days old) were exposed to V. cholerae in freshwater microcosms at four different cell densities (10^6^, 10^7^, 10^8^, and 10^9^ cells/mL). Six clinical V. cholerae O1 strains were tested, including the classical strains of the Ogawa (E7946) and Inaba (C6706 and N16961) serotypes and the Haitian variants (Table 1). Survival of larvae was unaffected by V. cholerae exposure (Fig. 1) until the highest cell density (10^9^ cells/mL) was reached, indicative of a threshold effect. At 10^9^ cells/mL, all six strains triggered significant larval mortality, with hazard ratios (HRs) ranging from 19 (strain E7946) to 10 (strain AA142) (Fig. 1B; see also Table S1 in the supplemental material). No significant strain variation was detected, suggesting that the V. cholerae strains had comparable influences on triggering chironomid mortality at this critically high cell density (*P* > 0.05 [Table S2]).

**TABLE 1:** Strains used in this study

Strain	Source and origin (location)	Yr of isolation	Serotype
E7946	Clinical, isolated from a cholera patient, Bahrain	1978	O1 El Tor Ogawa
AA142	Clinical, isolated from a cholera patient, Haiti	2010	O1 El Tor Ogawa
HC1037	Clinical, isolated from a cholera patient, Haiti	2014	O1 El Tor Ogawa
HC16	Clinical, isolated from a cholera patient, Haiti	2012	O1 El Tor Ogawa
C6706	Clinical, isolated from a cholera patient, Peru	1991	O1 El Tor Inaba
N16961	Clinical, isolated from a cholera patient, Bangladesh	1971	O1 El Tor Inaba

For 10^6^ to 10^8^ cells/mL, our data showed that V. cholerae exposure was generally nonpathogenic and in some cases beneficial. HRs were 1 order of magnitude lower at cell densities below 10^9^ cells/mL (Fig. 1B). More than 70% of the larvae survived through the course of 7 days across all treatment groups. The 95% confidence intervals of HRs of all strains covered 1 or were lower, indicating a potential survival benefit (Fig. 1C; Table S1). Intriguingly, at 10^8^ cells/mL, some V. cholerae strains (E7946, AA142, and HC1037) significantly enhanced larval survival compared to the control (Fig. 1C).

### V. cholerae prevalence in chironomid larvae correlates with inoculation dose.

**FIG 2:**
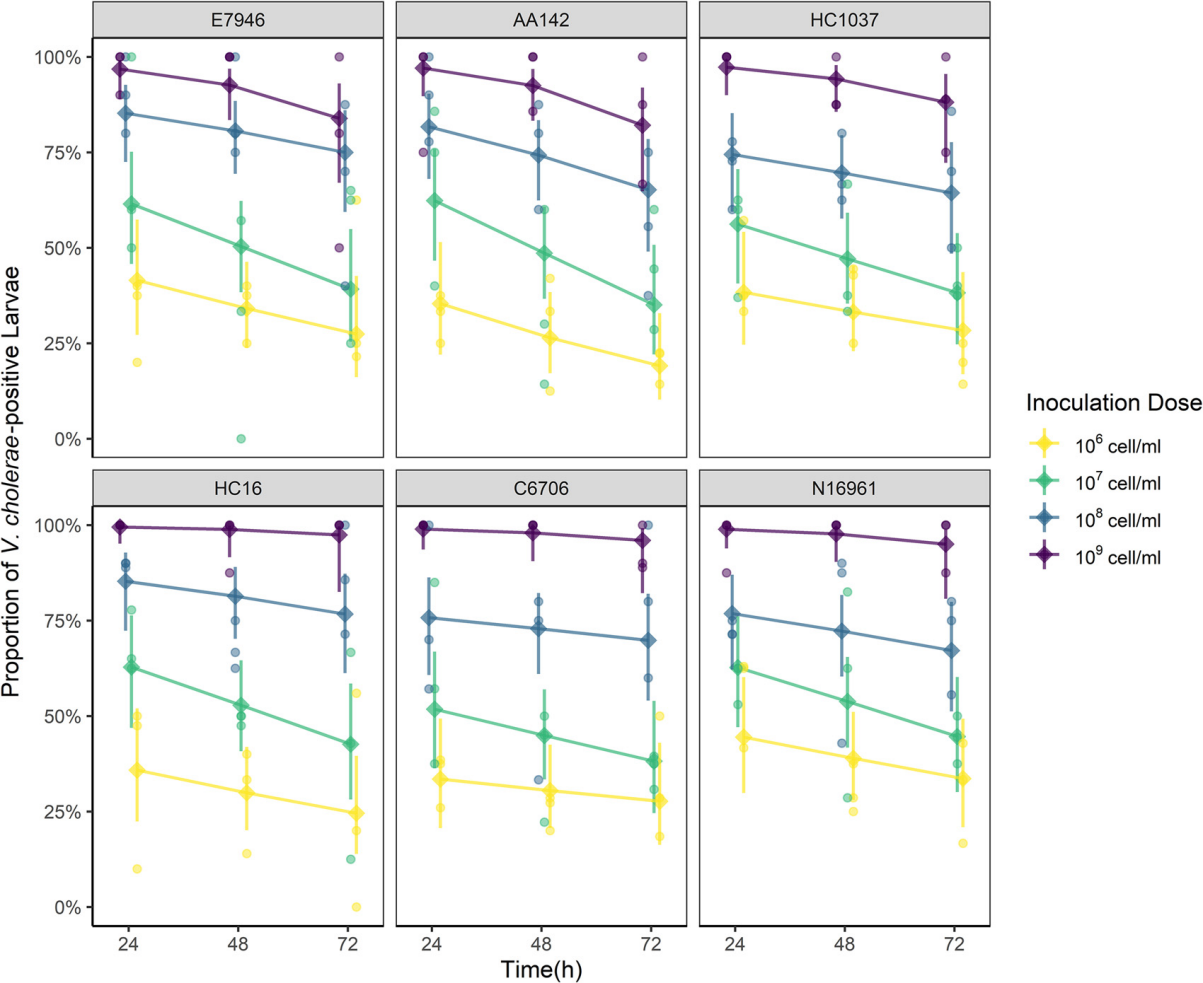
Invasion and prevalence of V. cholerae in the chironomid larvae. The proportion of V. cholerae-positive chironomid larvae (prevalence) after exposure to six different strains at different cell densities (10^6^, 10^7^, 10^8^, and 10^9^ cells/mL) was assayed every 24 h for 72 h (*n* = 16 microcosm wells of single larvae per treatment; the assay was repeated for three times).

The distinct survival outcomes between chironomid larvae exposed to 10^9^ cells/mL and those exposed to the lower cell densities could be attributed to differences in the invasion efficiency of V. cholerae at different doses. To test this, the proportion of V. cholerae-positive larvae was scored every 24 h for 72 h to determine V. cholerae prevalence over time. A logistic regression model was used to analyze the relationships between V. cholerae strain, inoculation dose, time, and prevalence. We observed a positive correlation between inoculation dose and prevalence (Fig. 2). Fractions of V. cholerae-positive larvae reached saturation when exposed to the bacterium at the high cell density (10^9^ cells/mL), persisting through the 72-h window (Fig. 2). In contrast, fewer than half of the larvae exposed to 10^6^ cells/mL of V. cholerae became positive. The odds ratios (ORs) of successful invasion at 10^7^, 10^8^, and 10^9^ cells/mL relative to 10^6^ cells/mL were 2.60 (*P* = 0.06), 8.25 (*P* < 0.001), and 75.22 (*P* < 0.001), respectively (Table S3). The time and strain effects were not significant (OR = 0.99; *P* > 0.05 [Table S3]), signifying that the prevalence of larvae carrying V. cholerae remained stable within the 72-h window and V. cholerae persistence did not differ among the strains.

### V. cholerae load in chironomid larvae correlates with inoculation dose and can vary by strain.

**FIG 3:**
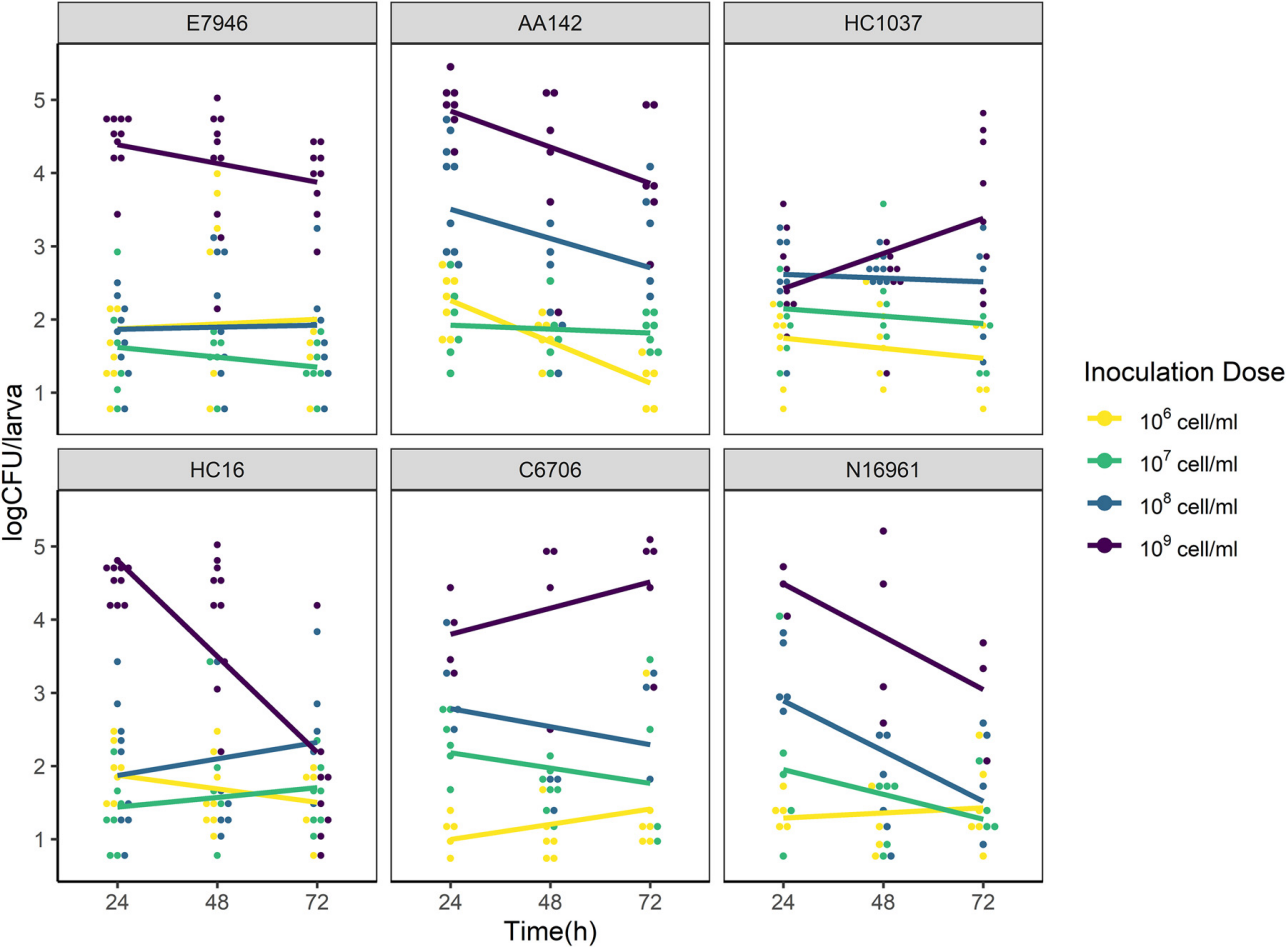
V. cholerae CFU in chironomid larvae after exposure. Chironomid larvae were exposed to V. cholerae in freshwater microcosms at four different cell densities (10^6^, 10^7^, 10^8^, and 10^9^ cells/mL). The CFU numbers per larva (log transformed) were assayed every 24 h for 72 h, and those with CFU number larger than 0 were included in the following analysis (*n* = 20 to 32 microcosm wells of single larvae per treatment).

Focusing on the V. cholerae-positive larvae, a linear model was fitted to explain how V. cholerae load (CFU numbers) varies by strain, time, and inoculation dose. The analysis showed that V. cholerae load in the chironomid larvae correlated positively with inoculation dose (F ratio = 233.54; *P* < 0.001) (Fig. 3). Interstrain variation in temporal CFU changes in the larvae was evident but also dependent on cell density, as supported by significant strain-inoculation dose interactions (*P* < 0.001 [Table S5]). Specifically, at 10^6^ and 10^7^ cells/mL, the V. cholerae CFU numbers remained stable throughout the 72-h window, except for strain AA142 at 10^6^ cells/mL, which exhibited a significant decline (β = −0.02; *P* < 0.01 [Table S6]). At densities above 10^7^ cells/mL, we observed notable interstrain differences in V. cholerae persistence, with some strains declining markedly (by up to 2 orders of magnitude) in CFU from 24 h to 48 or 72 h (strains AA142 and N16961 at 10^8^ and 10^9^ cells/mL and HC16 at 10^9^ cells/mL), while other strains remained relatively stable (Fig. 3; Table S6) and one strain showed an increase (HC1037 at 10^9^ cells/mL; β = 0.02; *P* < 0.01).

Overall, our data suggest that V. cholerae prevalence and load in larvae correlate positively with cell density, unlike the threshold effect observed in larval mortality. Furthermore, increasing V. cholerae prevalence and load from 10^6^ to 10^8^ cells/mL did not result in mortality, and some strains even promoted survival. Based on this observation, we conclude that interstrain variation in V. cholerae load did not predict larval survivorship.

### V. cholerae persistence in water varies depending on the strain, inoculation dose, and chironomid presence but does not predict invasion efficiency in chironomids.

**FIG 4:**
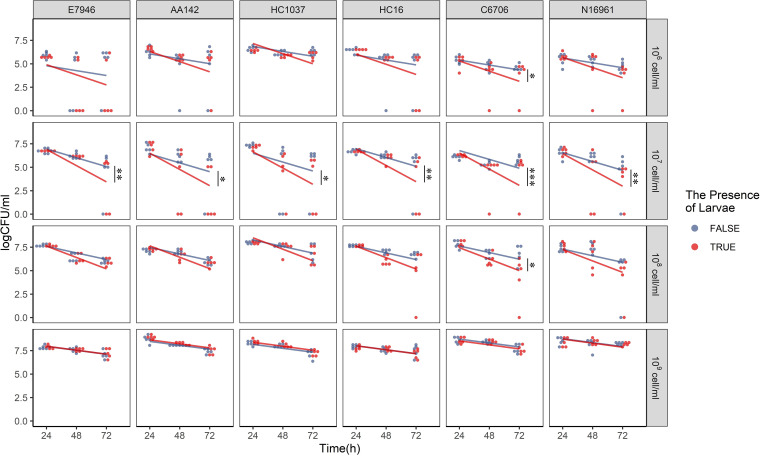
Temporal dynamics of V. cholerae CFU in the water. Freshwater microcosms containing chironomid larvae and without larvae were inoculated with single V. cholerae strains at four different cell densities (10^6^, 10^7^, 10^8^, and 10^9^ cells/mL). V. cholerae CFU in the water were sampled every 24 h for 72 h. A linear model was fitted for the whole data set. The regression lines generated from the model (red, microcosms with larvae; blue, without larvae) were plotted. The statistical significance of difference between CFU per milliliter in microcosms with and without larvae is shown (*, *P* < 0.05; **, *P* < 0.01; ***, *P* < 0.001).

We next asked whether (i) the presence of chironomid larvae affects the cell density of free-living V. cholerae in the water and (ii) variability in V. cholerae invasion dynamics of the chironomids reflects temporal abundance changes of V. cholerae in water. Quantifying V. cholerae CFU in the water with or without chironomid larvae every 24 h for 72 h, a linear model was fitted to test the effects of strain, inoculation dose, and larval presence on V. cholerae persistence in water. As shown in Fig. 4, we detected in some instances that the presence of larvae significantly dampened V. cholerae persistence in water (all strains at 10^7^ cells/mL and strain C6706 at 10^6^ and 10^8^ cells/mL). Yet we observed minimal fluctuation at the 10^9^-cell/mL inoculation dose. Together with the significant inoculation dose-larval presence (F ratio = 2.90; *P* < 0.05) and time-larval presence (F ratio = 13.94; *P* < 0.001) interactions, these findings lead us to conclude that the presence of larvae can accelerate the decline of free-living V. cholerae in the water when initial cell density is lower than 10^9^ cells/mL.

To test how the free-living V. cholerae CFU numbers correspond to the CFU numbers inside chironomid larvae, we performed a linear regression; the results suggested that there was a positive correlation (*R*^2^ = 0.35; *P* < 0.001 [Fig. S1]) between free-living and host (chironomid)-associated CFU numbers when taking all the samples across the four doses together. However, when we analyzed the data for each inoculation dose separately, the V. cholerae loads in the chironomid did not correlate with V. cholerae CFU number in the water (Fig. S2). The results indicate that V. cholerae persistence in water could not explain variability in V. cholerae load in the chironomids.

### V. cholerae exposure can reduce species evenness of the chironomid microbiome.

**FIG 5:**
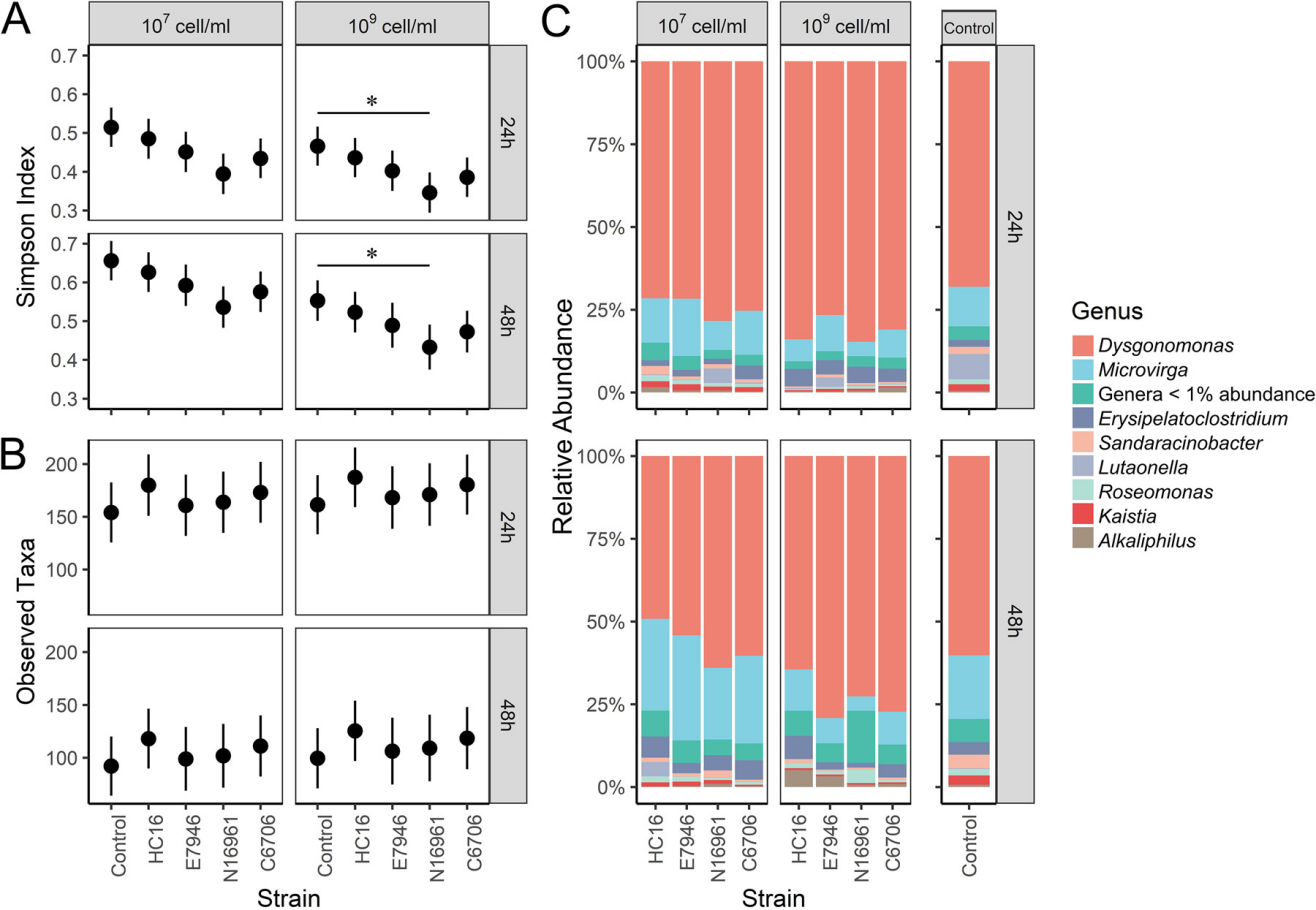
Microbiome diversity and composition in V. cholerae-exposed and control (unexposed) chironomid larvae at 24 h and 48 h, revealed by 16S rRNA gene amplicon sequencing. (A) Simpson index; (B) observed taxa. The estimated marginal mean of each index and its corresponding 95% confidence intervals are shown for each treatment group. The pairwise comparison between alpha diversity index of V. cholerae exposed larvae and control (unexposed) larvae is indicated (*, *P* < 0.05). Information for other alpha diversity indices can be found in Fig. S2. (C) Relative abundances of bacterial components detected by 16S rRNA gene amplicon sequencing. Each bar represents a biological replicate sample. Genera that account for less than 1% of the total abundance are grouped together and summarized as “Genera < 1% abundance.”

Our high-throughput sequencing data show that the chironomid larval microbiome was dominated by eight genera (Fig. 5C). Specifically, in unexposed larvae, *Dysgonomonas* had the highest relative abundance, accounting for 26.8 to 94.1% of the reads, followed by *Microvirga* (1.4 to 51.7% of the reads). We also noticed a large numbers of operational taxonomic units (OTUs) at <1% abundance in the samples that collectively made up 0.6 to 33.6% of the reads. To explore the impact of V. cholerae exposure on the chironomid microbiome, we tested four V. cholerae strains of different geographical origins at two cell densities (10^7^ and 10^9^ cells/mL). Our data show that V. cholerae exposure induced subtle yet notable changes on the microbiome composition over the course of 48 h. In terms of alpha diversity, the abundance-based coverage estimators (ACE, Chao1 index, and observed taxa) were unaffected, and the phylogenetic diversity (PD) was also not affected by the exposure (Fig. 5B; Fig. S9). However, the Simpson indices were reduced in larvae exposed to 10^9^ cells/mL, particularly for strain N16961 at both 24 h and 48 h (Fig. 5A; Table S9). The reduced Simpson indices but not observed taxa suggest that V. cholerae exposure reduces species evenness of the microbiome, which is supported by the slight increases in the dominant *Dysgonomonas* but declines in several low-abundance genera (e.g., *Microbacterium* and *Ancylobacter*). The linear discriminant analysis effect size (LEfSe) identified few taxa that marked the microbiome changes induced by specific V. cholerae strains and they were all at low abundance (<1%) in the unexposed larvae (Fig. S4). Beta diversity plots by principal-coordinate analysis (PCoA) based on the Bray-Curtis and Jaccard matrices with permutational multivariate analysis of variance (PERMANOVA) indicated weak clustering by strain (Fig. S5) at 10^9^ cells/mL, while no significant clustering was detected at a low concentration. These results suggest that V. cholerae exposure perturbs the chironomid microbiome by depleting some minor taxa, and this effect may be more general rather than strain specific.

## DISCUSSION

This study represents an important first step toward developing an experimental tractable system to study V. cholerae interactions with aquatic arthropods. Using freshwater microcosms housed with the chironomid larvae, we dissected the effects of aquatic cell density and strain on V. cholerae-chironomid interactions by varying the two parameters independently. We experimentally demonstrated that chironomid larvae can be exposed to V. cholerae without any detrimental effects up to a very high dose, regardless of the strain. Meanwhile, interstrain variability in host invasion, including prevalence, bacterial load, and effect on host survival, was found to be highly dose dependent. These findings illustrate that aquatic cell density is a key driver of V. cholerae invasion success of the chironomid larvae, while some strains may be better at persisting in chironomids or promoting survival of invaded hosts under specific cell density conditions. Interestingly, strains that are equally persistent in water at a given dose may mediate different carrying loads and persistence in chironomids, suggesting that interstrain variation in invasion dynamics is not predicted by water persistence. Increased persistence in the chironomid hosts may confer a fitness advantage on V. cholerae in the environment, both in survival (the chironomids may shield the bacterium from fluctuating water conditions and other environmental stressors) and in dissemination (chironomids may help transmit the bacterium to animals higher in the food chain). Together, our observations hint that V. cholerae environmental persistence may not solely be determined by how well the bacterium survives in water but also be contingent on its interactions with neighboring aquatic hosts.

Our study also provides insights on the interactions between V. cholerae and the chironomid microbiome. The microbial taxa identified in our chironomid larvae are generally consistent with the bacteria found in aquatic insects. *Dysgonomonas* was shown to be present in both laboratory and wild chironomids and account for about 2% of the total microbial abundance in lab-cultured chironomid egg masses (24). Interestingly, the relative abundance of *Dysgonomonas* in lab-reared larvae was found to be much higher than in the wild (25). This suggests that the laboratory conditions may favor chironomid-associated *Dysgonomonas*, which may explain its high abundance in our samples. *Dysgonomonas* is also frequently reported to be associated with larval mosquitoes, which also occupy diverse aquatic habitats. In the larvae of Aedes triseriatus, *Dysgonomonas* was shown to be the dominant genus, accounting for over 50% of the microbiome (26). However, *Dysgonomonas* was not found in A. triseriatus adults, suggesting this genus may be specific to the aquatic life stage. *Microvirga*, another major genus detected in our chironomid larvae, was previously shown to be a dominant bacterium in the larvae of Aedes aegypti and Anopheles coluzzii (27, 28). *Roseomonas* was also previously found at low abundance in chironomid larvae and *Aedes* and *Anopheles* mosquitos (both larvae and adults) (29, 30). Other genera detected in our chironomids include *Erysipelatoclostridium* and *Kaistia*, both of which have also been found in terrestrial insects such as lepidopterans, although their association with aquatic insects has not been described (31, 32).

Our results show that exposure to the four V. cholerae strains of different geographical origins (HC16, E7946, N16961, and C6706) did not change the species richness or phylogenetic diversity of the chironomid microbiome. Instead, larvae exposed at the high cell density exhibited a reduction in species evenness. The observation appears to be different from recent findings in zebrafish, wherein V. cholerae exposure leads to a notable reduction in microbiome species richness and a slight reduction in species evenness (33). It is important to note that zebrafish suffer diarrhea-like symptoms when exposed to V. cholerae at 10^5^- to 10^7^-CFU/mL cell densities, but in chironomid larvae, we did not detect signs of morbidity at doses below 10^9^ CFU/mL (34). In fact, our chironomid larvae exposed to V. cholerae at the lower doses developed into adults with success equal to that of the unexposed control (data not shown). An interesting inquiry for future investigation is whether microbiome resilience or perturbation patterns in response to V. cholerae may explain the distinct virulence outcomes in different hosts. Nonetheless, patterns of microbiome perturbation similar to those observed in our study have been described for several insect-microbe vector relationships. For example, the microbiome of the Asian citrus psyllid Diaphorina citri showed a reduced Shannon index upon infection by “*Candidatus* Liberibacter asiaticus,” a citrus plant pathogen (35). Similarly, the microbiome in triatomines *Triatoma* spp. did not change drastically when infected by the pathogen Trypanosoma cruzi (36). These observations suggest that the homeostasis of the microbiome in insects may play an important role in insect-pathogen interaction, which could be vital for insects to remain healthy.

While our study has shed light on V. cholerae-chironomid interactions in the context of cell density and strain, other abiotic factors, such as water temperature and salinity, can also influence the microbial dynamics and warrant future investigation. For example, V. cholerae has an optimum temperature of around 37°C and grows well under warmer conditions (37). In contrast, elevated temperatures have been shown to disrupt the microbiomes in a wide range of aquatic organisms, causing compositional shifts and depletion of low-abundance taxa (38). As water temperatures continue to rise due to global climate change, it would be interesting to test whether increasing water temperatures would exacerbate V. cholerae persistence and spread by promoting its growth while reducing resilience of aquatic microbiomes to pathogen invasion (39). Similarly, future work will include using the microcosms to infer how freshwater salinization (e.g., due to sea level rise), nutrient pulses (e.g., due to contamination of human or animal wastes), and drought may affect V. cholerae interactions with aquatic arthropods and their microbiomes and ultimately its aquatic persistence.

Another important area for future investigation concerns the effectors and mechanisms underlying V. cholerae colonization in aquatic arthropods. There is a consensus that V. cholerae gains nutritional benefits by attaching to biotic surfaces, such as chitinous surfaces of aquatic crustaceans, because they can utilize chitin as a carbon and nitrogen source (40, 41) and may, in turn, contribute to nutrient recycling (42). In this study, however, we specifically focused on V. cholerae inside chironomid hosts using surface-sterilized larvae. Intestinal colonization of V. cholerae has been extensively studied in mammalian models, most notably in rabbit and infant mouse models (43–48). These studies have focused on how V. cholerae withstands the gut biochemical environment, penetrates the mucus layer, and attaches to the epithelium. Many factors contributing to V. cholerae colonization and pathogenesis have been identified through these models. For example, cholera toxin-coregulated pili (TCP; type IV pili) have been shown to promote gut V. cholerae colonization and bacterial aggregation (49–51), and biofilm formation has been shown to enhance V. cholerae’s resistance to acidic pH (52) and antimicrobials (53, 54). The type VI secretion system has been shown to aid in competition against the host native gut microbiota, facilitating gene acquisition from other microbes (55, 56), and horizontal gene transfer (57). Additional genes and processes governing V. cholerae’s colonization include the lipopolysaccharide O-antigen (58), RND efflux systems (59), and flagellum-mediated motility (60). Model organisms, including Drosophila melanogaster and zebrafish, have also been used to identify V. cholerae gut colonization factors, yielding a high-throughput outcome (34, 61–63). However, despite V. cholerae’s natural association with aquatic arthropods, the molecular determinants underlying bacterial colonization and persistence in aquatic arthropods remain elusive (64). V. cholerae likely employs different strategies to evade the barriers unique to aquatic arthropods. For instance, the chironomid larval gut microenvironment has a neutral to slightly alkaline pH, in contrast to the acidic guts or stomachs in flies and mammals (65). Some of the preferred carbon sources of V. cholerae that are abundant in the mammalian mucus layers, including specific glycans like sialic acid (*N*-acetylneuraminic acid) and GlcNAc (*N*-acetylglucosamine), are absent or differentially expressed in arthropod guts.

It is worth noting that there are several limitations to our study. First, while the dose range we tested aligns with the inoculation doses used in V. cholerae studies on other nonmammalian host models (56, 66, 67), these doses are likely higher than V. cholerae cell densities in the field, which often range from 10 to 10^5^ cell/mL (34, 63, 66, 68–70). However, significantly higher densities (up to 10^14^ CFU/mL) have also been reported in regions of cholera endemicity with heavy human activity, such as the Dhaleshwari River in Bangladesh and aquaculture ponds (71). Additionally, some scientists believe that V. cholerae is more abundant than estimated due to its ability to transition into the viable-but-nonculturable (VBNC) state under unfavorable field conditions, where it remains metabolically active but does not grow on standard microbiological media (72–74).

Second, 16S rRNA gene amplicon sequencing of our samples detected only a few V. cholerae reads. To investigate further, we performed quantitative PCR (qPCR) using V. cholerae-specific (OmpW) and general 16S rRNA gene primers, and the results suggested that V. cholerae abundance was low relative to the chironomid microbiome (data not shown). We hypothesized that the limited detection of V. cholerae by amplicon sequencing was due to the method’s known tendency to preferentially amplify abundant taxa over rare ones, a phenomenon known as species masking (75–77). This bias can be exacerbated by the presence of a hyperabundant taxon (*Dysgonomonas* across our samples), which may have contributed to the poor detection of V. cholerae reads.

Third, this study focused exclusively on clinical V. cholerae strains of the O1 and O139 serogroups that can cause diarrheal disease in humans. There exists a vast diversity of environmental V. cholerae strains that are genetically distinct from the clinical strains and lack key virulence factors, including the cholera toxin (CTX) and TCP (78, 79). Some environmental strains are believed to contribute to V. cholerae divergence, serving as a donor of genetic materials. Experiments investigating lower dose ranges and whether V. cholerae genomic variation affects its interactions with chironomid larvae are under way.

### Conclusion.

Our study provides the first demonstration that chironomids can be used as an experimental host system to study V. cholerae interactions with aquatic hosts. With the use of freshwater microcosms, this study has revealed the extent to which V. cholerae invasion and persistence in chironomid larvae, as well as chironomid response to V. cholerae exposure, vary by aquatic cell density and strain. This novel system provides a robust, cost-effective approach to help identify the diverse strategies V. cholerae employs to enable its survival under changing conditions and can generate results that potentially mimic the interactions occurring in the natural aquatic environment.

## MATERIALS AND METHODS

### Chironomid collection and rearing.

Wild chironomid larvae were collected from experimental potholes at the USDA Agricultural Research Service station (29°38′09.6″N, 82°21′43.0″W) in Gainesville, FL, USA, to establish a colony in the laboratory. The colony was maintained at room temperature, under a 12-h:12-h light-dark cycle, in metal rearing cages (30 by 30 by 30 cm) with nylon netting. Each cage was connected to a plastic tray (36 by 27 by 14 cm) containing 13 L of aged tap water (tap water exposed to air for more than 7 days) with aeration and an opening at the top (15 by 15 cm). The water was changed weekly with 2 g of finely ground fish food (Tetra, USA) per tray. Adult chironomids were provided with a 10% (vol/vol) honey-water solution. Egg masses were collected from the water once per week using clean forceps and then transferred to separate plastic trays (7 cm in diameter) with 50 mL of aged tap water. Hatched larvae were then transferred back to the colonies. The chironomid species was identified as *Chironomus columbiensis* by sequencing of the COI gene using primers LCO1490 (5′-GGTCAACAAATCATAAAGATATTGG-3′) and HCO2198 (5′-TAAACTTCAGGGTGACCAAAAAATCA-3′).

### Vibrio cholerae strains and inoculation experiments.

Details of Vibrio cholerae strains used in the experiments are described in Table 1. To avoid including bacteria other than V. cholerae in the chironomids when counting the CFU, antibiotic-resistant V. cholerae for each strain was generated. V. cholerae was first plated on LB plates with 50 μg/mL of streptomycin, and then the surviving colonies were picked and selected again with 200 μg/mL of streptomycin.

Inoculation experiments were conducted in freshwater microcosms in an arthropod containment level 2 (ACL-2) facility. Fresh lake water was first filtered using a test sieve (45 mm in mesh; VWR, USA), and then the flowthrough was further filtered using a 500-mL filtration unit (0.2 μm; Thermo Fisher Scientific, USA) and stored at 4°C until used. Absence of bacteria in the filter-sterilized lake water was confirmed as no visible colony formation after plating onto LB, MRS, and tryptic soy broth (TSB) (Fisher Scientific, USA) agar plates and incubation at 30°C overnight. To prepare a V. cholerae inoculum, an overnight culture of V. cholerae was set up to prepare a bacterial pellet, which then was washed and diluted to desired cell densities (10^6^ to 10^9^ CFU/mL) using filter-sterilized lake water. Microcosms were set up by transferring 2 mL of the inoculated lake water into individual wells of sterile 24-well plates (PALL, USA). Single chironomid larvae at the 4th-instar stage were placed into each well, alongside no-chironomid controls (Fig. 1).

### Assessment of V. cholerae prevalence and loads.

After placing individual late-instar larvae into the microcosms, V. cholerae prevalence and loads in the chironomid larvae and water were quantified at 24 h, 48 h, and 72 h postexposure. Only live larvae were picked for the following plating. Briefly, larvae were surface sterilized by rinsing two times in 0.5% (vol/vol) sodium hypochlorite-water solution for 1 min, followed by three times in sterile water for 1 min. Surface-sterilized larvae were homogenized in 500 μL of phosphate-buffered saline (PBS) using plastic grinding pestles (Capitol Scientific, USA) and then serially diluted for plating. For lake water samples, 10 μL of lake water in each well was serially diluted and plated. Both larval homogenate and water samples were plated on LB agar with 200 μg/mL of streptomycin overnight for V. cholerae enumeration. To assess the prevalence of V. cholerae, 16 microcosms were set for each treatment group and the assay was repeated three times. The detection of V. cholerae colonies was considered successful invasion. To assess the CFU numbers in larvae and water, 8 to 18 microcosms were set for each treatment group and the assay was repeated three times.

### Survival of chironomids following V. cholerae exposure.

Upon V. cholerae exposure, chironomid larval survival in the microcosms was monitored twice daily at 12-h intervals (7:30 a.m. and 7:30 p.m.). Chironomid larvae in lake water without V. cholerae were set up as a control. All treatment groups were run in 20 replicates.

### High-throughput sequencing of the 16S rRNA gene amplicons.

Chironomid larvae were collected at 24 h and 48 h upon V. cholerae exposure at two cell densities (10^7^ and 10^9^ cells/mL). Larvae in lake water without V. cholerae were set up as a control. Eight replicate samples were prepared per treatment group, each containing a single larva. The larvae were surface sterilized by rinsing two times in 0.5% (vol/vol) sodium hypochlorite-water solution for 1 min, followed by three times in sterile water for 1 min. Total genomic DNA was isolated using a DNeasy blood and tissue kit (Qiagen, Hilden, Germany) following the manufacturer’s instructions. DNA quantity and quality were assessed by a NanoDrop One instrument (Thermo Fisher Scientific, USA) and agarose gel electrophoresis. The precise concentration of DNA was further validated using a Qubit 4 fluorometer (Thermo Fisher Scientific). The hypervariable regions (V3-V4) of the bacterial 16S rRNA gene were chosen for amplicon sequencing. The DNA samples were sent to Novogene Corporation Inc. (Beijing, China) for library preparation and sequenced using an Illumina NovaSeq 6000 machine (Illumina, USA) with 2 × 250-bp paired-end (PE) chemistry.

### Amplicon sequence analysis.

Paired-end reads were first processed using FastQC 0.11.9 (80). The reads were then imported to the QIIME2 (2021.4) environment and demultiplexed (81). After quality checking, the reads were denoised and dereplicated, the chimeras were removed using Divisive Amplicon Denoising Algorithm 2 (DADA2) (82), and an operational taxonomic unit (OTU) table for OTU representative sequences was generated. Taxonomic assignments for OTUs were performed using q2-feature-classifier with the GreenGenes 13.08 database with a 99% identity (83). To exclude OTUs other than bacteria, we used the Basic Local Alignment Search Tool to query the representative OTU sequences against the National Center for Biotechnology Information (NCBI) NR database and excluded OTUs annotated to nonbacterial organisms.

### Statistical analysis.

All statistical analysis was performed in R 4.0.3 (84), and all the figures were generated using R package ggplot2 (85). The survival ratio was analyzed using the R package survminer 0.4.9 by fitting data into COX proportional-hazards (COX PH) model with Firth’s penalized likelihood, and the hazard ratio of each treatment was generated based on each model. Pair-wise comparison was then conducted using the log rank test (86). CFU data on chironomid larvae were log transformed and fixed into a linear model. The CFU among all treatment groups were compared pair-wise with Benjamini-Hochberg adjustment on estimated marginal means generated from the linear model, using R package emmeans 1.7.2 (87). CFU data on water were fixed into a mixed linear model by setting biological replicates as the mixed effect, using R package lme4 (88). The CFU among all treatment groups were compared pair-wise with Benjamini-Hochberg adjustment on estimated marginal means generated from the linear model, using R package emmeans 1.7.2 (87).

For amplicon sequencing data, QIIME2 output files were imported and analyzed in R using R package phyloseq (89). The ACE index, Chao1 index, observed taxa, phylogenetic diversity (PD), Simpson index, and Shannon index were calculated in R package vegan 2.5.7 to indicate the alpha diversity (90). A generalized linear model (ACE index, Chao1 index, and observed taxa) and linear model (PD, Simpson index, and Shannon index) were fitted to each index. For each index, pairwise comparison was conducted among all treatment groups with Benjamini-Hochberg adjustment on estimated marginal means generated from corresponding models using R package emmeans (87). The beta-diversity was calculated through principal-coordinate analysis (PCoA) using the Bray-Curtis matrix and Jaccard matrix. The statistics were analyzed using adonis analysis with 999 Monte Carlo permutations using R package vegan 2.5.7 (90). R package microbiomeMarker 3.15 was used to identify differentially abundant genera that were discriminant between clusters using linear discriminant analysis effect size (LEfSe) (91). Briefly, LEfSe analyzes the differentially distributed taxonomic units across sample clusters using a Kruskal-Wallis rank sum test. Next, the effect size of each differentially abundant taxon is estimated by building an linear discriminant analysis (LDA) model. Further, the taxonomic units that are discriminative with respect to clusters are ranked based on their effect size. The *P* value for LEfSe was set to 0.05, and the logarithmic LDA score cutoff was set to 2.0.

### Data availability.

Sequencing data supporting this study are openly available from GenBank at PRJNA862186 (16S rRNA gene amplicon sequencing) and OQ221601 (COI gene of *Chironomus columbiensis*).

## References

[1] Ali M, Nelson AR, Lopez AL, Sack DA. 2015. Updated global burden of cholera in endemic countries. PLoS Negl Trop Dis 9:e0003832. doi:10.1371/journal.pntd.0003832. 26043000 PMC4455997

[2] Chac D, Dunmire CN, Singh J, Weil AA. 2021. Update on environmental and host factors impacting the risk of Vibrio cholerae infection. ACS Infect Dis 7:1010–1019. doi:10.1021/acsinfecdis.0c00914. 33844507

[3] Reidl J, Klose KE. 2002. Vibrio cholerae and cholera: out of the water and into the host. FEMS Microbiol Rev 26:125–139. doi:10.1111/j.1574-6976.2002.tb00605.x. 12069878

[4] Weber GG, Kortmann J, Narberhaus F, Klose KE. 2014. RNA thermometer controls temperature-dependent virulence factor expression in Vibrio cholerae. Proc Natl Acad Sci USA 111:14241–14246. doi:10.1073/pnas.1411570111. 25228776 PMC4191814

[5] Nelson EJ, Harris JB, Glenn Morris J, Calderwood SB, Camilli A. 2009. Cholera transmission: the host, pathogen and bacteriophage dynamic. Nat Rev Microbiol 7:693–702. doi:10.1038/nrmicro2204. 19756008 PMC3842031

[6] Silva-Valenzuela CA, Camilli A. 2019. Niche adaptation limits bacteriophage predation of Vibrio cholerae in a nutrient-poor aquatic environment. Proc Natl Acad Sci USA 116:1627–1632. doi:10.1073/pnas.1810138116. 30635420 PMC6358685

[7] Colwell RR, Huq A. 1994. Disease in evolution: global changes and emergence of infectious diseases. Ann N Y Acad Sci 740:44–54. doi:10.1111/j.1749-6632.1994.tb19852.x. 7840440

[8] Lutz C, Erken M, Noorian P, Sun S, McDougald D. 2013. Environmental reservoirs and mechanisms of persistence of Vibrio cholerae. Front Microbiol 4:375. doi:10.3389/fmicb.2013.00375. 24379807 PMC3863721

[9] Huq A, West PA, Small EB, Huq MI, Colwell RR. 1984. Influence of water temperature, salinity, and pH on survival and growth of toxigenic Vibrio cholerae serovar 01 associated with live copepods in laboratory microcosms. Appl Environ Microbiol 48:420–424. doi:10.1128/aem.48.2.420-424.1984. 6486784 PMC241529

[10] Nahar S, Sultana M, Naser MN, Nair GB, Watanabe H, Ohnishi M, Yamamoto S, Endtz H, Cravioto A, Sack RB, Hasan NA, Sadique A, Huq A, Colwell RR, Alam M. 2011. Role of shrimp chitin in the ecology of toxigenic Vibrio cholerae and cholera transmission. Front Microbiol 2:260. doi:10.3389/fmicb.2011.00260. 22319512 PMC3250921

[11] Dalia AB, Lazinski DW, Camilli A. 2014. Identification of a membrane-bound transcriptional regulator that links chitin and natural competence in Vibrio cholerae. mBio 5:e01028-13. doi:10.1128/mBio.01028-13. 24473132 PMC3903286

[12] Jubair M, Morris JG, Ali A. 2012. Survival of Vibrio cholerae in nutrient-poor environments is associated with a novel “persister” phenotype. PLoS One 7:e45187. doi:10.1371/journal.pone.0045187. 23028836 PMC3445476

[13] Schuhmacher DA, Klose KE. 1999. Environmental signals modulate ToxT-dependent virulence factor expression in Vibrio cholerae. J Bacteriol 181:1508–1514. doi:10.1128/JB.181.5.1508-1514.1999. 10049382 PMC93540

[14] Prouty MG, Klose KE. 2014. Vibrio cholerae: the genetics of pathogenesis and environmental persistence, p 309–339. *In* Thompson FL, Austin B, Swings J (ed), The biology of vibrios. ASM Press, Washington, DC.

[15] Mavian C, Paisie TK, Alam MT, Browne C, Beau De Rochars VM, Nembrini S, Cash MN, Nelson EJ, Azarian T, Ali A, Morris JG, Salemi M. 2020. Toxigenic Vibrio cholerae evolution and establishment of reservoirs in aquatic ecosystems. Proc Natl Acad Sci USA 117:7897–7904. doi:10.1073/pnas.1918763117. 32229557 PMC7149412

[16] Broza M, Gancz H, Kashi Y. 2008. The association between non-biting midges and Vibrio cholerae. Environ Microbiol 10:3193–3200. doi:10.1111/j.1462-2920.2008.01714.x. 19025555

[17] Laviad-Shitrit S, Sela R, Thorat L, Sharaby Y, Izhaki I, Nath BB, Halpern M. 2020. Identification of chironomid species as natural reservoirs of toxigenic Vibrio cholerae strains with pandemic potential. PLoS Negl Trop Dis 14:e0008959. doi:10.1371/journal.pntd.0008959. 33362241 PMC7757795

[18] Almagro-Moreno S, Taylor RK. 2013. Cholera: environmental reservoirs and impact on disease transmission. Microbiol Spectr 1:OH-0003-2012. doi:10.1128/microbiolspec.OH-0003-2012. 26184966

[19] Broza M, Halpern M. 2001. Chironomid egg masses and Vibrio cholerae. Nature 412:40. doi:10.1038/35083691. 11452294

[20] Raz N, Danin-Poleg Y, Broza YY, Arakawa E, Ramakrishna BS, Broza M, Kashi Y. 2010. Environmental monitoring of Vibrio cholerae using chironomids in India: chironomid exuviae as a monitoring tool of V. cholerae. Environ Microbiol Rep 2:96–103. doi:10.1111/j.1758-2229.2009.00109.x. 23766003

[21] Kuncham R, Sivaprakasam T, Puneeth Kumar R, Sreenath P, Nayak R, Thayumanavan T, Subba Reddy GV. 2017. Bacterial fauna associating with chironomid larvae from lakes of Bengaluru city, India—a 16s rRNA gene based identification. Genom Data 12:44–48. doi:10.1016/j.gdata.2017.03.001. 28316932 PMC5342978

[22] Camacho A, Bouhenia M, Alyusfi R, Alkohlani A, Naji MAM, de Radiguès X, Abubakar AM, Almoalmi A, Seguin C, Sagrado MJ, Poncin M, McRae M, Musoke M, Rakesh A, Porten K, Haskew C, Atkins KE, Eggo RM, Azman AS, Broekhuijsen M, Saatcioglu MA, Pezzoli L, Quilici M-L, Al-Mesbahy AR, Zagaria N, Luquero FJ. 2018. Cholera epidemic in Yemen, 2016–18: an analysis of surveillance data. Lancet Glob Health 6:e680–e690. doi:10.1016/S2214-109X(18)30230-4. 29731398 PMC5952990

[23] Rieckmann A, Tamason CC, Gurley ES, Rod NH, Jensen PKM. 2018. Exploring droughts and floods and their association with cholera outbreaks in Sub-Saharan Africa: a register-based ecological study from 1990 to 2010. Am J Trop Med Hyg 98:1269–1274. doi:10.4269/ajtmh.17-0778. 29512484 PMC5953376

[24] Laviad-Shitrit S, Sela R, Sharaby Y, Thorat L, Nath BB, Halpern M. 2021. Comparative microbiota composition across developmental stages of natural and laboratory-reared Chironomus circumdatus populations from India. Front Microbiol 12:746830. doi:10.3389/fmicb.2021.746830. 34899634 PMC8661057

[25] Sela R, Laviad-Shitrit S, Thorat L, Nath BB, Halpern M. 2021. Chironomus ramosus larval microbiome composition provides evidence for the presence of detoxifying enzymes. Microorganisms 9:1571. doi:10.3390/microorganisms9081571. 34442650 PMC8398091

[26] Juma EO, Allan BF, Kim C-H, Stone C, Dunlap C, Muturi EJ. 2021. The larval environment strongly influences the bacterial communities of Aedes triseriatus and Aedes japonicus (Diptera: Culicidae). Sci Rep 11:7910. doi:10.1038/s41598-021-87017-0. 33846445 PMC8042029

[27] Hery L, Guidez A, Durand A-A, Delannay C, Normandeau-Guimond J, Reynaud Y, Issaly J, Goindin D, Legrave G, Gustave J, Raffestin S, Breurec S, Constant P, Dusfour I, Guertin C, Vega-Rúa A. 2021. Natural variation in physicochemical profiles and bacterial communities associated with Aedes aegypti breeding sites and larvae on Guadeloupe and French Guiana. Microb Ecol 81:93–109. doi:10.1007/s00248-020-01544-3. 32621210 PMC7794107

[28] Krajacich BJ, Huestis DL, Dao A, Yaro AS, Diallo M, Krishna A, Xu J, Lehmann T. 2018. Investigation of the seasonal microbiome of Anopheles coluzzii mosquitoes in Mali. PLoS One 13:e0194899. doi:10.1371/journal.pone.0194899. 29596468 PMC5875798

[29] Tchouassi DP, Muturi EJ, Arum SO, Kim C-H, Fields CJ, Torto B. 2019. Host species and site of collection shape the microbiota of Rift Valley fever vectors in Kenya. PLoS Negl Trop Dis 13:e0007361. doi:10.1371/journal.pntd.0007361. 31173595 PMC6584011

[30] Muturi EJ, Ramirez JL, Rooney AP, Kim C-H. 2017. Comparative analysis of gut microbiota of mosquito communities in central Illinois. PLoS Negl Trop Dis 11:e0005377. doi:10.1371/journal.pntd.0005377. 28245239 PMC5345876

[31] Higuita Palacio MF, Montoya OI, Saldamando CI, García-Bonilla E, Junca H, Cadavid-Restrepo GE, Moreno-Herrera CX. 2021. Dry and rainy seasons significantly alter the gut microbiome composition and reveal a key Enterococcus sp. (Lactobacillales: Enterococcaceae) core component in Spodoptera frugiperda (Lepidoptera: Noctuidae) corn strain from Northwestern Colombia. J Insect Sci 21:10. doi:10.1093/jisesa/ieab076. PMC8567080 34734290

[32] Oliveira NC, Rodrigues PAP, Cônsoli FL. 2022. Host-adapted strains of Spodoptera frugiperda hold and share a core microbial community across the Western Hemisphere. Microb Ecol doi:10.1007/s00248-022-02008-6. 35426077

[33] Breen P, Winters AD, Theis KR, Withey JH. 2021. Vibrio cholerae infection induces strain-specific modulation of the zebrafish intestinal microbiome. Infect Immun 89:e00157-21. doi:10.1128/IAI.00157-21. 34061623 PMC8370672

[34] Runft DL, Mitchell KC, Abuaita BH, Allen JP, Bajer S, Ginsburg K, Neely MN, Withey JH. 2014. Zebrafish as a natural host model for Vibrio cholerae colonization and transmission. Appl Environ Microbiol 80:1710–1717. doi:10.1128/AEM.03580-13. 24375135 PMC3957598

[35] Song X, Peng A, Ling J, Cui Y, Cheng B, Zhang L. 2019. Composition and change in the microbiome of Diaphorina citri infected with Candidatus Liberibacter asiaticus in China. Int J Trop Insect Sci 39:283–290. doi:10.1007/s42690-019-00036-3.

[36] Mann AE, Mitchell EA, Zhang Y, Curtis-Robles R, Thapa S, Hamer SA, Allen MS. 2020. Comparison of the bacterial gut microbiome of North American Triatoma spp. with and without Trypanosoma cruzi. Front Microbiol 11:364. doi:10.3389/fmicb.2020.00364. 32231645 PMC7082358

[37] Froelich BA, Daines DA. 2020. In hot water: effects of climate change on Vibrio–human interactions. Environ Microbiol 22:4101–4111. doi:10.1111/1462-2920.14967. 32114705

[38] Sehnal L, Brammer-Robbins E, Wormington AM, Blaha L, Bisesi J, Larkin I, Martyniuk CJ, Simonin M, Adamovsky O. 2021. Microbiome composition and function in aquatic vertebrates: small organisms making big impacts on aquatic animal health. Front Microbiol 12:567408. doi:10.3389/fmicb.2021.567408. 33776947 PMC7995652

[39] Trinanes J, Martinez-Urtaza J. 2021. Future scenarios of risk of Vibrio infections in a warming planet: a global mapping study. Lancet Planet Health 5:e426–e435. doi:10.1016/S2542-5196(21)00169-8. 34245713

[40] Meibom KL, Li XB, Nielsen AT, Wu C-Y, Roseman S, Schoolnik GK. 2004. The Vibrio cholerae chitin utilization program. Proc Natl Acad Sci USA 101:2524–2529. doi:10.1073/pnas.0308707101. 14983042 PMC356983

[41] Blokesch M. 2012. Chitin colonization, chitin degradation and chitin-induced natural competence of Vibrio cholerae are subject to catabolite repression. Environ Microbiol 14:1998–1912. doi:10.1111/j.1462-2920.2011.02689.x. 22222000

[42] Pruzzo C, Vezzulli L, Colwell RR. 2008. Global impact of Vibrio cholerae interactions with chitin: V. cholerae-chitin interactions. Environ Microbiol 10:1400–1410. doi:10.1111/j.1462-2920.2007.01559.x. 18312392

[43] Duan F, March JC. 2010. Engineered bacterial communication prevents Vibrio cholerae virulence in an infant mouse model. Proc Natl Acad Sci USA 107:11260–11264. doi:10.1073/pnas.1001294107. 20534565 PMC2895089

[44] Fu Y, Waldor MK, Mekalanos JJ. 2013. Tn-Seq analysis of Vibrio cholerae intestinal colonization reveals a role for T6SS-mediated antibacterial activity in the host. Cell Host Microbe 14:652–663. doi:10.1016/j.chom.2013.11.001. 24331463 PMC3951154

[45] Abel S, Waldor MK. 2015. Infant rabbit model for diarrheal diseases. Curr Protoc Microbiol 38:6A.6.1–6A.6.15. doi:10.1002/9780471729259.mc06a06s38. PMC5084528 26237109

[46] Alavi S, Mitchell JD, Cho JY, Liu R, Macbeth JC, Hsiao A. 2020. Interpersonal gut microbiome variation drives susceptibility and resistance to cholera infection. Cell 181:1533–1546.e13. doi:10.1016/j.cell.2020.05.036. 32631492 PMC7394201

[47] Sikora AE. 2018. Vibrio cholerae: methods and protocols. Springer, New York, NY.

[48] Sit B, Fakoya B, Waldor MK. 2022. Animal models for dissecting Vibrio cholerae intestinal pathogenesis and immunity. Curr Opin Microbiol 65:1–7. doi:10.1016/j.mib.2021.09.007. 34695646 PMC8792189

[49] Clavijo AP, Bai J, Gómez-Duarte OG. 2010. The longus type IV pilus of enterotoxigenic Escherichia coli (ETEC) mediates bacterial self-aggregation and protection from antimicrobial agents. Microb Pathog 48:230–238. doi:10.1016/j.micpath.2010.03.006. 20227481 PMC2860038

[50] Krebs SJ, Taylor RK. 2011. Protection and attachment of Vibrio cholerae mediated by the toxin-coregulated pilus in the infant mouse model. J Bacteriol 193:5260–5270. doi:10.1128/JB.00378-11. 21804008 PMC3187450

[51] Echazarreta MA, Klose KE. 2019. Vibrio flagellar synthesis. Front Cell Infect Microbiol 9:131. doi:10.3389/fcimb.2019.00131. 31119103 PMC6504787

[52] Zhu J, Mekalanos JJ. 2003. Quorum sensing-dependent biofilms enhance colonization in Vibrio cholerae. Dev Cell 5:647–656. doi:10.1016/s1534-5807(03)00295-8. 14536065

[53] Gupta P, Mankere B, Chekkoora Keloth S, Tuteja U, Pandey P, Chelvam KT. 2018. Increased antibiotic resistance exhibited by the biofilm of Vibrio cholerae O139. J Antimicrob Chemother 73:1841–1847. doi:10.1093/jac/dky127. 29688490

[54] Merrell DS, Butler SM, Qadri F, Dolganov NA, Alam A, Cohen MB, Calderwood SB, Schoolnik GK, Camilli A. 2002. Host-induced epidemic spread of the cholera bacterium. Nature 417:642–645. doi:10.1038/nature00778. 12050664 PMC2776822

[55] Fast D, Kostiuk B, Foley E, Pukatzki S. 2018. Commensal pathogen competition impacts host viability. Proc Natl Acad Sci USA 115:7099–7104. doi:10.1073/pnas.1802165115. 29915049 PMC6142279

[56] Logan SL, Thomas J, Yan J, Baker RP, Shields DS, Xavier JB, Hammer BK, Parthasarathy R. 2018. The Vibrio cholerae type VI secretion system can modulate host intestinal mechanics to displace gut bacterial symbionts. Proc Natl Acad Sci USA 115:E3779–E3787. doi:10.1073/pnas.1720133115. 29610339 PMC5910850

[57] Borgeaud S, Metzger LC, Scrignari T, Blokesch M. 2015. The type VI secretion system of Vibrio cholerae fosters horizontal gene transfer. Science 347:63–67. doi:10.1126/science.1260064. 25554784

[58] Mukhopadhyay S, Nandi B, Ghose AC. 2000. Antibodies (IgG) to lipopolysaccharide of Vibrio cholerae O1 mediate protection through inhibition of intestinal adherence and colonisation in a mouse model. FEMS Microbiol Lett 185:29–35. doi:10.1111/j.1574-6968.2000.tb09036.x. 10731603

[59] Bina XR, Provenzano D, Nguyen N, Bina JE. 2008. Vibrio cholerae RND family efflux systems are required for antimicrobial resistance, optimal virulence factor production, and colonization of the infant mouse small intestine. Infect Immun 76:3595–3605. doi:10.1128/IAI.01620-07. 18490456 PMC2493215

[60] Lee SH, Butler SM, Camilli A. 2001. Selection for in vivo regulators of bacterial virulence. Proc Natl Acad Sci USA 98:6889–6894. doi:10.1073/pnas.111581598. 11391007 PMC34448

[61] Purdy AE, Watnick PI. 2011. Spatially selective colonization of the arthropod intestine through activation of Vibrio cholerae biofilm formation. Proc Natl Acad Sci USA 108:19737–19742. doi:10.1073/pnas.1111530108. 22106284 PMC3241763

[62] Nag D, Farr DA, Walton MG, Withey JH. 2020. Zebrafish models for pathogenic vibrios. J Bacteriol 202:e00165-20. doi:10.1128/JB.00165-20. 32778562 PMC7685555

[63] Kamareddine L, Wong ACN, Vanhove AS, Hang S, Purdy AE, Kierek-Pearson K, Asara JM, Ali A, Morris JG, Jr, Watnick PI. 2018. Activation of Vibrio cholerae quorum sensing promotes survival of an arthropod host. Nat Microbiol 3:243–252. doi:10.1038/s41564-017-0065-7. 29180725 PMC6260827

[64] Espinoza-Vergara G, Noorian P, Silva-Valenzuela CA, Raymond BBA, Allen C, Hoque MM, Sun S, Johnson MS, Pernice M, Kjelleberg S, Djordjevic SP, Labbate M, Camilli A, McDougald D. 2019. Vibrio cholerae residing in food vacuoles expelled by protozoa are more infectious in vivo. Nat Microbiol 4:2466–2474. doi:10.1038/s41564-019-0563-x. 31570868 PMC7071789

[65] Stief P, Eller G. 2006. The gut microenvironment of sediment-dwelling Chironomus plumosus larvae as characterised with O2, pH, and redox microsensors. J Comp Physiol B 176:673–683. doi:10.1007/s00360-006-0090-y. 16721623

[66] Blow NS, Salomon RN, Garrity K, Reveillaud I, Kopin A, Jackson FR, Watnick PI. 2005. Vibrio cholerae infection of Drosophila melanogaster mimics the human disease cholera. PLoS Pathog 1:e8. doi:10.1371/journal.ppat.0010008. 16201020 PMC1238743

[67] Kaito C, Akimitsu N, Watanabe H, Sekimizu K. 2002. Silkworm larvae as an animal model of bacterial infection pathogenic to humans. Microb Pathog 32:183–190. doi:10.1006/mpat.2002.0494. 12079408

[68] Franco AA, Fix AD, Prada A, Paredes E, Palomino JC, Wright AC, Johnson JA, McCarter R, Guerra H, Morris JG. 1997. Cholera in Lima, Peru, correlates with prior isolation of Vibrio cholerae from the environment. Am J Epidemiol 146:1067–1075. doi:10.1093/oxfordjournals.aje.a009235. 9420531

[69] Schauer S, Sommer R, Farnleitner AH, Kirschner AKT. 2012. Rapid and sensitive quantification of Vibrio cholerae and Vibrio mimicus cells in water samples by use of catalyzed reporter deposition fluorescence in situ hybridization combined with solid-phase cytometry. Appl Environ Microbiol 78:7369–7375. doi:10.1128/AEM.02190-12. 22885749 PMC3457089

[70] Nasreen T, Hussain NAS, Ho JY, Aw VZJ, Alam M, Yanow SK, Boucher YF. 2022. Assay for evaluating the abundance of Vibrio cholerae and its O1 serogroup subpopulation from water without DNA extraction. Pathogens 11:363. doi:10.3390/pathogens11030363. 35335687 PMC8953119

[71] Real M, Khanam N, Mia M, Nasreen M. 2017. Assessment of water quality and microbial load of Dhaleshwari River Tangail, Bangladesh. Adv Microbiol 7:523–533. doi:10.4236/aim.2017.76041.

[72] Cottingham KL, Chiavelli DA, Taylor RK. 2003. Environmental microbe and human pathogen: the ecology and microbiology of Vibrio cholerae. Front Ecol Environ 1:80–86. doi:10.1890/1540-9295(2003)001[0080:EMAHPT]2.0.CO;2.

[73] Sun H, Zhu C, Fu X, Khattak S, Wang J, Liu Z, Kong Q, Mou H, Secundo F. 2022. Effects of intestinal microbiota on physiological metabolism and pathogenicity of Vibrio. Front Microbiol 13:947767. doi:10.3389/fmicb.2022.947767. 36081796 PMC9445811

[74] Zhao X, Zhong J, Wei C, Lin C-W, Ding T. 2017. Current perspectives on viable but non-culturable state in foodborne pathogens. Front Microbiol 8:580. doi:10.3389/fmicb.2017.00580. 28421064 PMC5378802

[75] Skelton J, Cauvin A, Hunter ME. 2022. Environmental DNA metabarcoding read numbers and their variability predict species abundance, but weakly in non-dominant species. Environ DNA doi:10.1002/edn3.355.

[76] Wilcox TM, Zarn KE, Piggott MP, Young MK, McKelvey KS, Schwartz MK. 2018. Capture enrichment of aquatic environmental DNA: a first proof of concept. Mol Ecol Resour 18:1392–1401. doi:10.1111/1755-0998.12928. 30009542

[77] Gonzalez JM, Portillo MC, Belda-Ferre P, Mira A. 2012. Amplification by PCR artificially reduces the proportion of the rare biosphere in microbial communities. PLoS One 7:e29973. doi:10.1371/journal.pone.0029973. 22253843 PMC3256211

[78] Schwartz K, Hammerl JA, Göllner C, Strauch E. 2019. Environmental and clinical strains of Vibrio cholerae non-O1, non-O139 from Germany possess similar virulence gene profiles. Front Microbiol 10:733. doi:10.3389/fmicb.2019.00733. 31031724 PMC6474259

[79] Dalsgaard A, Serichantalergs O, Forslund A, Lin W, Mekalanos J, Mintz E, Shimada T, Wells JG. 2001. Clinical and environmental isolates of Vibrio cholerae serogroup O141 carry the CTX phage and the genes encoding the toxin-coregulated pili. J Clin Microbiol 39:4086–4092. doi:10.1128/JCM.39.11.4086-4092.2001. 11682534 PMC88491

[80] Andrews S. 2010. FastQC: a quality control tool for high throughput sequence data. http://www.bioinformatics.babraham.ac.uk/projects/fastqc/.

[81] Bolyen E, Rideout JR, Dillon MR, Bokulich NA, Abnet CC, Al-Ghalith GA, Alexander H, Alm EJ, Arumugam M, Asnicar F, Bai Y, Bisanz JE, Bittinger K, Brejnrod A, Brislawn CJ, Brown CT, Callahan BJ, Caraballo-Rodríguez AM, Chase J, Cope EK, Da Silva R, Diener C, Dorrestein PC, Douglas GM, Durall DM, Duvallet C, Edwardson CF, Ernst M, Estaki M, Fouquier J, Gauglitz JM, Gibbons SM, Gibson DL, Gonzalez A, Gorlick K, Guo J, Hillmann B, Holmes S, Holste H, Huttenhower C, Huttley GA, Janssen S, Jarmusch AK, Jiang L, Kaehler BD, Kang KB, Keefe CR, Keim P, Kelley ST, Knights D, et al. 2019. Reproducible, interactive, scalable and extensible microbiome data science using QIIME 2. Nat Biotechnol 37:852–857. doi:10.1038/s41587-019-0209-9. 31341288 PMC7015180

[82] Callahan BJ, McMurdie PJ, Rosen MJ, Han AW, Johnson AJA, Holmes SP. 2016. DADA2: high-resolution sample inference from Illumina amplicon data. Nat Methods 13:581–583. doi:10.1038/nmeth.3869. 27214047 PMC4927377

[83] DeSantis TZ, Hugenholtz P, Larsen N, Rojas M, Brodie EL, Keller K, Huber T, Dalevi D, Hu P, Andersen GL. 2006. Greengenes, a chimera-checked 16S rRNA gene database and workbench compatible with ARB. Appl Environ Microbiol 72:5069–5072. doi:10.1128/AEM.03006-05. 16820507 PMC1489311

[84] R Core Team. 2018. R: a language and environment for statistical computing. R Foundation for Statistical Computing, Vienna, Austria. https://www.R-project.org/.

[85] Wickham H. 2016. ggplot2: Elegant Graphics for Data Analysis. Springer-Verlag New York. https://ggplot2.tidyverse.org.

[86] Kassambara A, Kosinski M, Biecek P. 2021. survminer: drawing survival curves using “ggplot2.” https://CRAN.R-project.org/package=survminer.

[87] Lenth RV. 2022. emmeans: estimated marginal means, aka least-squares means. https://CRAN.R-project.org/package=emmeans.

[88] Bates D, Mächler M, Bolker B, Walker S. 2014. Fitting linear mixed-effects models using lme4. arXiv 1406.5823 [stat.CO]. doi:10.48550/arXiv.1406.5823.

[89] McMurdie PJ, Holmes S. 2013. phyloseq: an R package for reproducible interactive analysis and graphics of microbiome census data. PLoS One 8:e61217. doi:10.1371/journal.pone.0061217. 23630581 PMC3632530

[90] Oksanen J, Blanchet FG, Friendly M, Kindt R, Legendre P, McGlinn D, Minchin PR, O’Hara RB, Simpson GL, Solymos P, Stevens MHH, Szoecs E, Wagner H. 2020. vegan: community ecology package. https://CRAN.R-project.org/package=vegan.

[91] Cao Y, Dong Q, Wang D, Zhang P, Liu Y, Niu C. 2022. microbiomeMarker: an R/Bioconductor package for microbiome marker identification and visualization. Bioinformatics doi:10.1093/bioinformatics/btac438. 35771644

