# Supplementary material for: Vibrio cholerae Invasion Dynamics of the Chironomid Host Are Strongly Influenced by Aquatic Cell Density and Can Vary by Strain
Source: Microbiol Spectr. 2023 Apr 19;11(3):e02652-22. doi: 10.1128/spectrum.02652-22 (PMC10269514; doi:10.1128/spectrum.02652-22)
Supplement: Supplemental file 1 — Fig. S1 to S5. Download spectrum.02652-22-s0001.pdf, PDF file, 1.1 MB [file spectrum.02652-22-s0001.pdf]

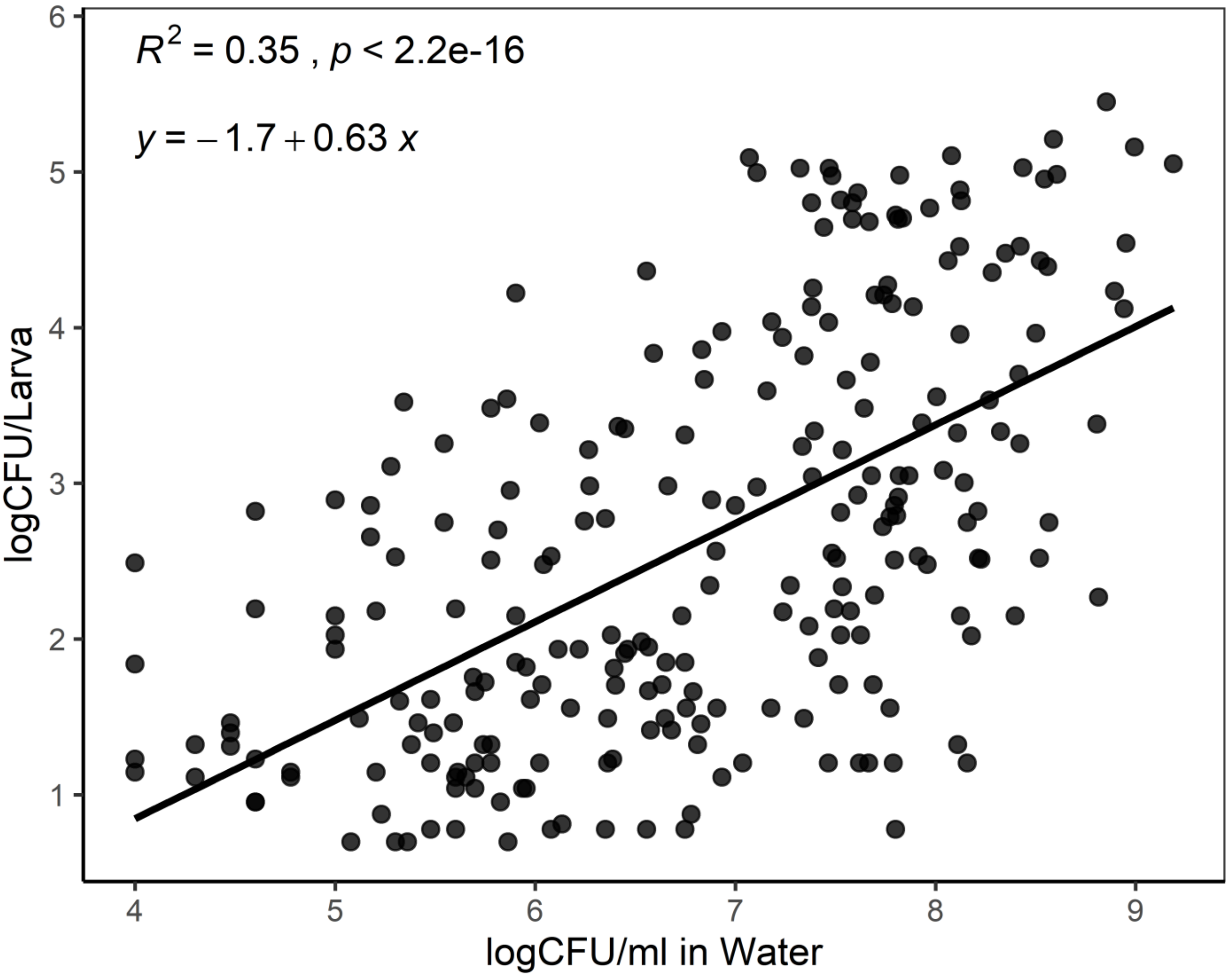

Figure S1. Relationships between free-living and host (chironomid)-associated *V. cholerae* CFUs. Linear regression of *V. cholerae* CFUs in the chironomid larvae and in water was conducted by pooling the data from all inoculation does groups together. Only larvae successfully invaded by *V. cholerae* (CFU > 0) were included in the analysis.

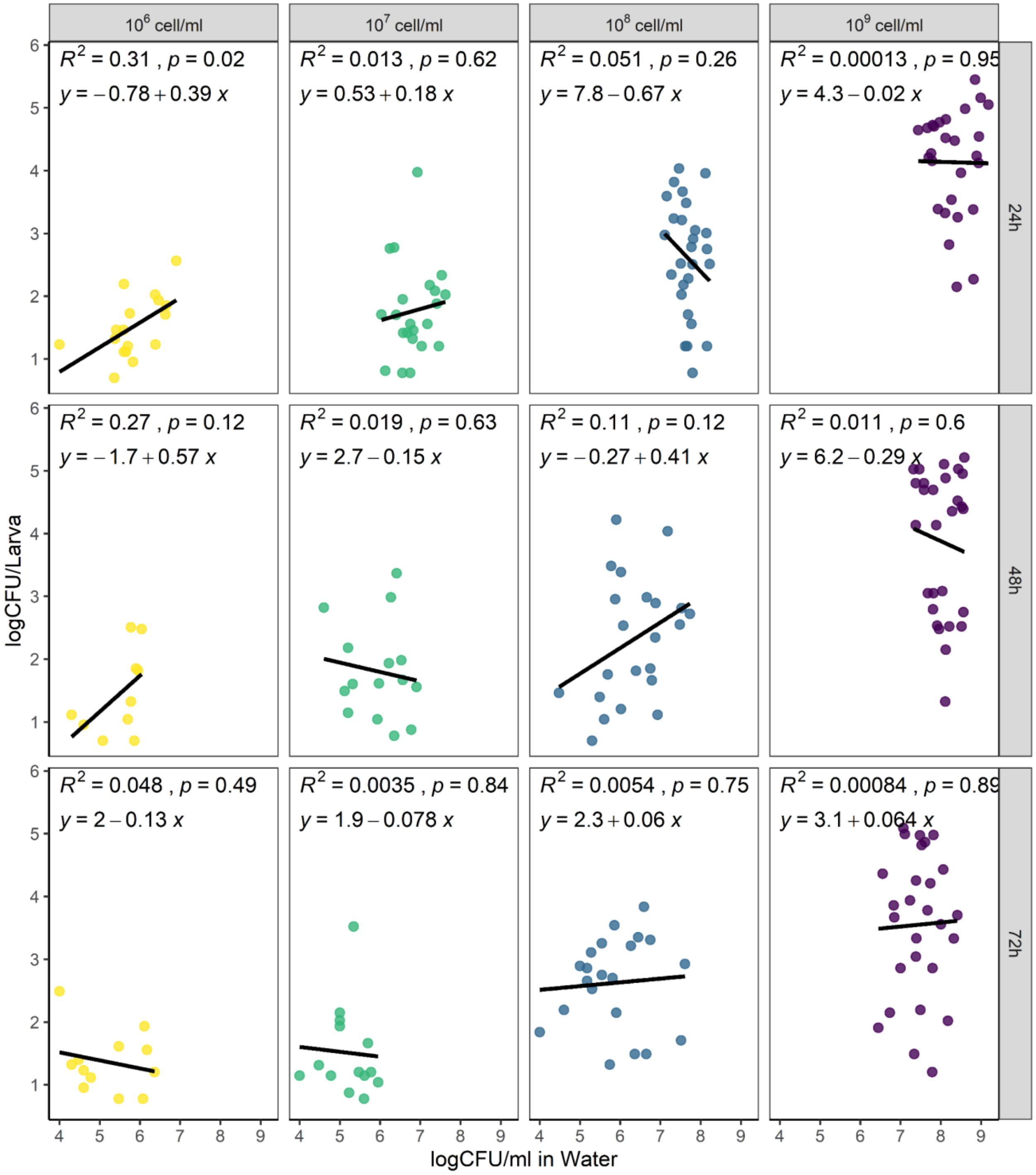

Figure S2. Relationships between free-living and host (chironomid)-associated *V. cholerae* CFUs at different inoculation doses. Linear regression was conducted separately at given inoculation doses to test the relationship between *V. cholerae* CFUs in the chironomid larvae and in water. Only larvae successfully invaded by *V. cholerae* (CFU > 0) were included in the analysis.

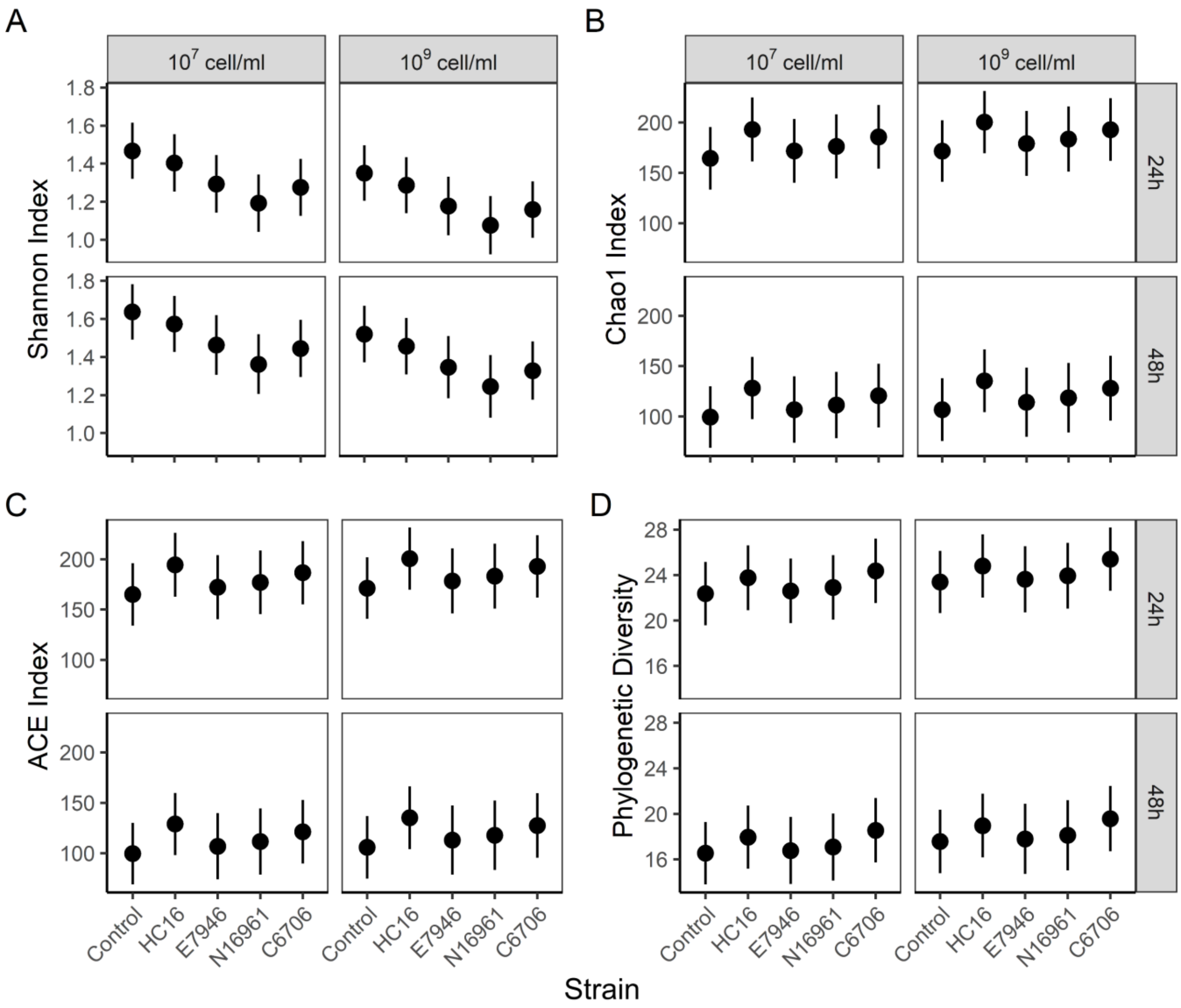

Figure S3. Microbiome alpha diversity of *V. cholerae*-exposed and control (unexposed) chironomid larvae. (A) Shannon Index; (B) Chao1 Index; (C) Ace Index; (D) Phylogenetic Diversity (PD); Estimated marginal mean of each index and its corresponding 95% confidence intervals were shown for each treatment group.

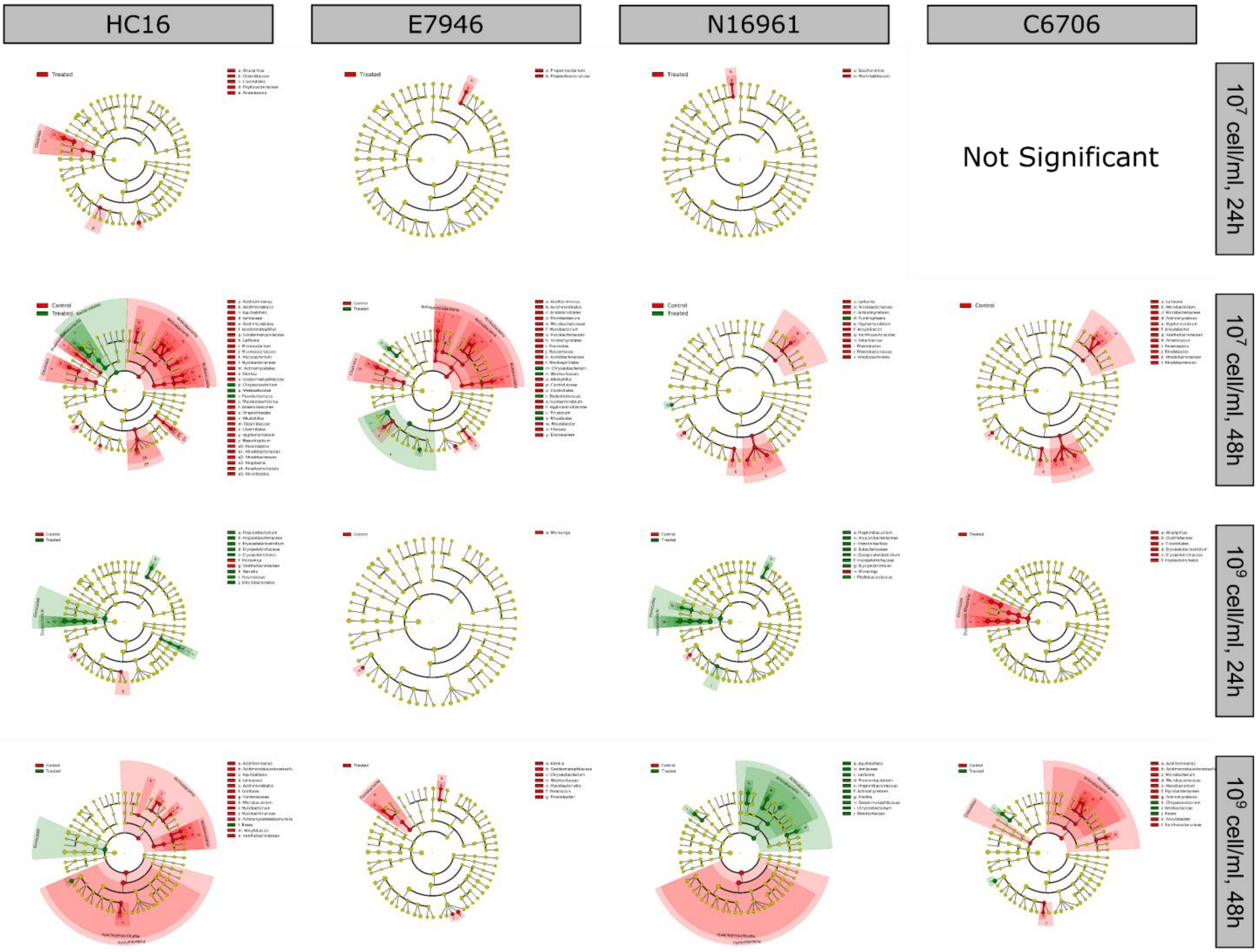

Figure S4. Phylum through genus taxonomic cladogram representation of LEfSe analysis of 16S rRNA gene sequences. Different colors indicate control group and *V. cholerae* exposed groups.

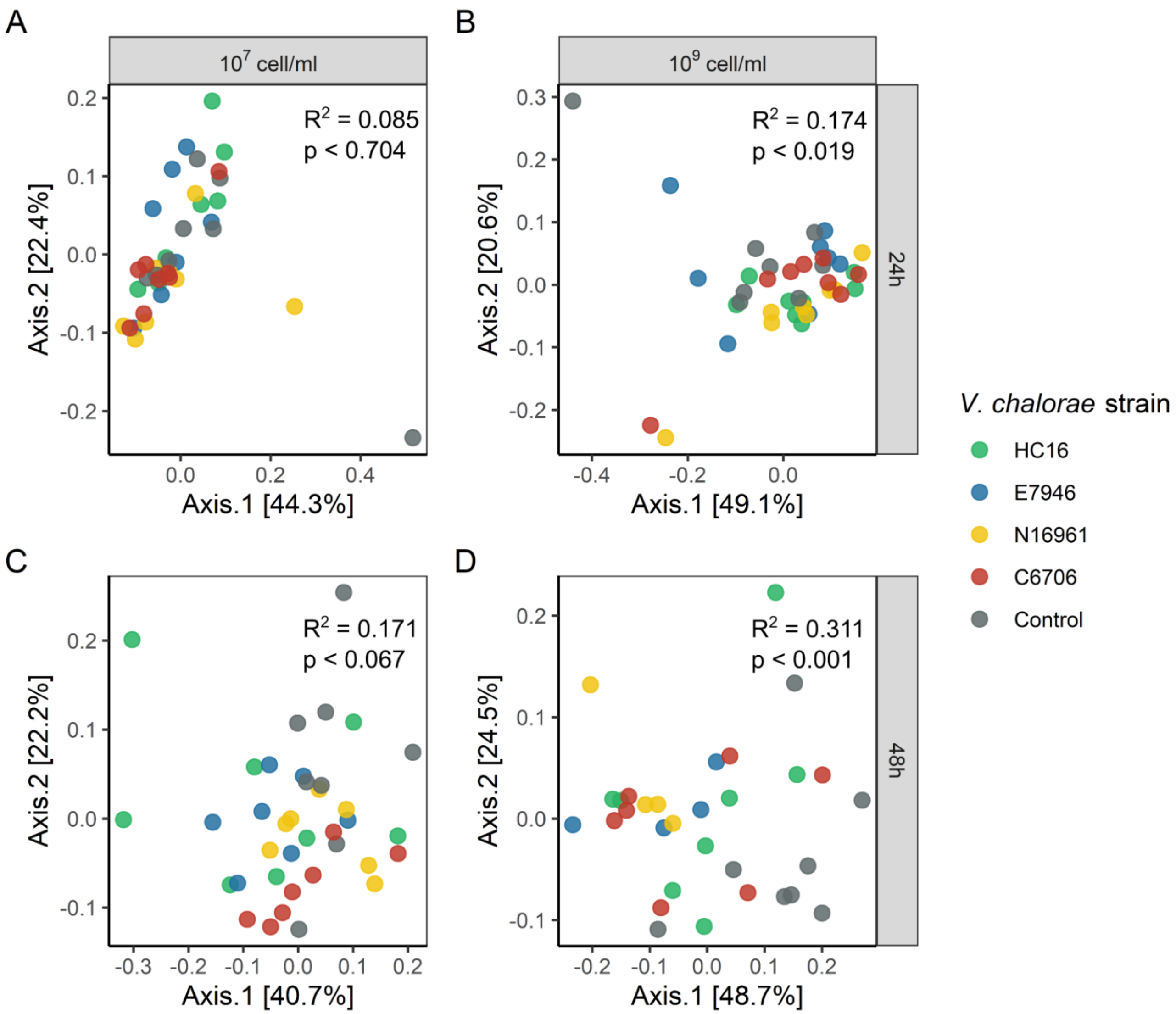

Figure S5. Microbiome beta diversity of *V. cholerae*-exposed and control (unexposed) chironomid larvae. Bray-Curtis principal components analysis (PCoA) plots of bacterial communities. The significant differences in beta diversities were analyzed using Adonis analysis with 999 Monte Carlo permutations.
