# Supplementary material for: Vibrio cholerae Invasion Dynamics of the Chironomid Host Are Strongly Influenced by Aquatic Cell Density and Can Vary by Strain
Source: Microbiol Spectr. 2023 Apr 19;11(3):e02652-22. doi: 10.1128/spectrum.02652-22 (PMC10269514; doi:10.1128/spectrum.02652-22)
Supplement: Supplemental file 2 — Tables S1 to S9. Download spectrum.02652-22-s0002.docx, DOCX file, 0.06 MB [file spectrum.02652-22-s0002.docx]

Table S1. Hazard Ration

| Strain | Cell Density (cell/ml) | Hazard Ratio | lower 95% confidence interval | upper 95% confidence interval | Chi-square | p Value |
| --- | --- | --- | --- | --- | --- | --- |
| E7946 | 10^6^ | 0.2575391 | 0.02778517 | 1.09536 | 3.31245736 | 0.06875661 |
| AA142 | 10^6^ | 0.4639798 | 0.08952333 | 1.599898 | 1.37655572 | 0.24068871 |
| HC1037 | 10^6^ | 0.8361395 | 0.24713545 | 2.393739 | 0.1029567 | 0.74830992 |
| HC16 | 10^6^ | 0.4441477 | 0.08567419 | 1.532204 | 1.54776345 | 0.21346592 |
| C6706 | 10^6^ | 0.76166 | 0.22526551 | 2.178684 | 0.24128743 | 0.6232778 |
| N16961 | 10^6^ | 0.9282976 | 0.30499278 | 2.522091 | 0.02009641 | 0.88726815 |
| E7946 | 10^7^ | 0.2578489 | 0.02782358 | 1.096235 | 3.307837239 | 0.06895018 |
| AA142 | 10^7^ | 0.4166084 | 0.08033691 | 1.437977 | 1.818860715 | 0.17744895 |
| HC1037 | 10^7^ | 0.6949171 | 0.17544442 | 2.138766 | 0.367944667 | 0.54412674 |
| HC16 | 10^7^ | 0.4050275 | 0.07807323 | 1.398919 | 1.944531946 | 0.16317731 |
| C6706 | 10^7^ | 1.5311131 | 0.5746392 | 3.881632 | 0.768900577 | 0.38055738 |
| N16961 | 10^7^ | 1.0059724 | 0.29710777 | 2.88293 | 0.000111145 | 0.99158845 |
| E7946 | 10^8^ | 0.07476274 | 0.00058105 | 0.5810306 | 7.20E+00 | 0.007298432 |
| AA142 | 10^8^ | 0.08246141 | 0.000641127 | 0.6396708 | 6.50E+00 | 0.01076284 |
| HC1037 | 10^8^ | 0.11883408 | 0.000923498 | 0.9239665 | 4.24E+00 | 0.039386167 |
| HC16 | 10^8^ | 0.43343143 | 0.083602613 | 1.4953899 | 1.65E+00 | 0.199140391 |
| C6706 | 10^8^ | 0.72827304 | 0.21513168 | 2.0864467 | 3.28E-01 | 0.566832795 |
| N16961 | 10^8^ | 0.99795871 | 0.328038613 | 2.7097651 | 1.51E-05 | 0.996903258 |
| E7946 | 10^9^ | 19.29242 | 8.64026 | 46.14612 | 52.93021 | 3.46E-13 |
| AA142 | 10^9^ | 10.02497 | 4.553867 | 23.60011 | 33.73999 | 6.30E-09 |
| HC1037 | 10^9^ | 16.90096 | 7.489983 | 40.82227 | 47.65448 | 5.08E-12 |
| HC16 | 10^9^ | 14.90627 | 6.589524 | 36.09939 | 43.52614 | 4.18E-11 |
| C6706 | 10^9^ | 16.81129 | 7.40048 | 40.84366 | 46.83188 | 7.73E-12 |
| N16961 | 10^9^ | 11.21255 | 5.212735 | 25.50333 | 37.72592 | 8.14E-10 |

p value adjustment method: Benjamini-Hochberg

Table S2. Log Rank Test for Survival Assay

| 10^6^ cell/ml |  |  |  |  |  |
| --- | --- | --- | --- | --- | --- |
| p Value | E7946 | AA142 | HC1037 | HC16 | C6706 |
| AA142 | 0.782320632 | NA | NA | NA | NA |
| HC1037 | 0.782320632 | 0.782320632 | NA | NA | NA |
| HC16 | 0.782320632 | 0.955339611 | 0.782320632 | NA | NA |
| C6706 | 0.782320632 | 0.782320632 | 0.955339611 | 0.782320632 | NA |
| N16961 | 0.782320632 | 0.782320632 | 0.941183375 | 0.782320632 | 0.929587952 |
| 10^7^ cell/ml |  |  |  |  |  |
| p Value | E7946 | AA142 | HC1037 | HC16 | C6706 |
| AA142 | 0.670855079 | NA | NA | NA | NA |
| HC1037 | 0.553291079 | 0.670855079 | NA | NA | NA |
| HC16 | 0.670855079 | 0.942394475 | 0.670855079 | NA | NA |
| C6706 | 0.210458495 | 0.210458495 | 0.467119506 | 0.210458495 | NA |
| N16961 | 0.363455944 | 0.467119506 | 0.670855079 | 0.467119506 | 0.670855079 |
| 10^8^ cell/ml |  |  |  |  |  |
| p Value | E7946 | AA142 | HC1037 | HC16 | C6706 |
| AA142 | 1 | NA | NA | NA | NA |
| HC1037 | 1 | 1 | NA | NA | NA |
| HC16 | 0.279552111 | 0.306053462 | 0.410196917 | NA | NA |
| C6706 | 0.15206119 | 0.15206119 | 0.279552111 | 0.679905666 | NA |
| N16961 | 0.144503289 | 0.144503289 | 0.15206119 | 0.350654013 | 0.783081942 |
| 10^9^ cell/ml |  |  |  |  |  |
| p Value | E7946 | AA142 | HC1037 | HC16 | C6706 |
| AA142 | 0.094129284 | NA | NA | NA | NA |
| HC1037 | 0.954782328 | 0.094129284 | NA | NA | NA |
| HC16 | 0.869406026 | 0.141052658 | 0.869406026 | NA | NA |
| C6706 | 0.869406026 | 0.094129284 | 0.954782328 | 0.869406026 | NA |
| N16961 | 0.194117436 | 0.869406026 | 0.705377534 | 0.913128751 | 0.869406026 |

p value adjustment method: Bonferroni

Table S3. Odds Ratio of Invasion Rate

| Model Term | Odds Ratio | Lower 95% CI | Upper 95% CI | p Value |
| --- | --- | --- | --- | --- |
| Cell Density 10^7^ cell/ml | 2.589672341 | 0.956300345 | 7.012862507 | 0.062794595 |
| Cell Density 10^8^ cell/ml | 8.253186595 | 2.699522015 | 25.23227763 | 2.83E-04 |
| Cell Density 10^9^ cell/ml | 75.2175763 | 11.08555966 | 510.365192 | 1.67E-05 |
| Strain AA142 | 0.859032915 | 0.252547819 | 2.921971586 | 0.808068756 |
| Strain HC1037 | 0.802366681 | 0.239499113 | 2.68807797 | 0.721537188 |
| Strain HC16 | 0.755192226 | 0.2167951 | 2.630665074 | 0.659763764 |
| Strain C6706 | 0.594844135 | 0.173463862 | 2.039845885 | 0.409783203 |
| Strain N16961 | 1.038291734 | 0.30847659 | 3.494753763 | 0.951678509 |
| Time | 0.986989948 | 0.969961826 | 1.004317006 | 0.14197829 |

Table S4. Pairwise Comparison of Invasion Rate

| Contrast | Cell Density (cell/ml) | Estimate | Standard Error | z Statistics | p Value |
| --- | --- | --- | --- | --- | --- |
| AA142 vs N16961 | 10^6^ | -0.573343788 | 0.376813859 | -1.521557063 | 0.650307398 |
| HC16 vs N16961 | 10^6^ | -0.402408258 | 0.368278607 | -1.092673456 | 0.884521061 |
| C6706 vs N16961 | 10^6^ | -0.373352999 | 0.366487427 | -1.018733445 | 0.911872427 |
| E7946 vs AA142 | 10^6^ | 0.364769137 | 0.382068829 | 0.954721008 | 0.931983655 |
| AA142 vs HC1037 | 10^6^ | -0.322568051 | 0.382720614 | -0.842829049 | 0.959474416 |
| HC1037 vs N16961 | 10^6^ | -0.250775737 | 0.362673192 | -0.691464776 | 0.982954645 |
| E7946 vs N16961 | 10^6^ | -0.208574651 | 0.36203393 | -0.576119071 | 0.99259368 |
| E7946 vs HC16 | 10^6^ | 0.193833607 | 0.373727648 | 0.518649365 | 0.995468166 |
| AA142 vs C6706 | 10^6^ | -0.19999079 | 0.386388895 | -0.517589383 | 0.9955116 |
| E7946 vs C6706 | 10^6^ | 0.164778348 | 0.371996663 | 0.442956521 | 0.997857167 |
| AA142 vs HC16 | 10^6^ | -0.17093553 | 0.387983656 | -0.440574049 | 0.99791177 |
| HC1037 vs HC16 | 10^6^ | 0.151632521 | 0.374362633 | 0.405041817 | 0.998605744 |
| HC1037 vs C6706 | 10^6^ | 0.122577262 | 0.372620124 | 0.328960391 | 0.999491949 |
| E7946 vs HC1037 | 10^6^ | 0.042201086 | 0.368221759 | 0.114607801 | 0.999997251 |
| HC16 vs C6706 | 10^6^ | -0.029055259 | 0.378076077 | -0.076850299 | 0.999999626 |
| C6706 vs N16961 | 10^7^ | -0.357576224 | 0.350593361 | -1.019917269 | 0.911469419 |
| HC16 vs C6706 | 10^7^ | 0.318276399 | 0.350834229 | 0.907198822 | 0.944816515 |
| HC1037 vs N16961 | 10^7^ | -0.268702663 | 0.350785924 | -0.766001839 | 0.973167457 |
| HC1037 vs HC16 | 10^7^ | -0.229402838 | 0.351027181 | -0.653518733 | 0.98678786 |
| E7946 vs C6706 | 10^7^ | 0.218523957 | 0.351091148 | 0.622413747 | 0.989422478 |
| AA142 vs N16961 | 10^7^ | -0.208794342 | 0.353127026 | -0.591272619 | 0.99164757 |
| AA142 vs HC16 | 10^7^ | -0.169494517 | 0.353367213 | -0.479655471 | 0.996868291 |
| AA142 vs C6706 | 10^7^ | 0.148781882 | 0.352676876 | 0.42186458 | 0.998304132 |
| E7946 vs N16961 | 10^7^ | -0.139052267 | 0.35153065 | -0.395562284 | 0.998756264 |
| E7946 vs HC1037 | 10^7^ | 0.129650396 | 0.351285538 | 0.369074106 | 0.999110678 |
| E7946 vs HC16 | 10^7^ | -0.099752443 | 0.351773472 | -0.283570111 | 0.999754441 |
| HC1037 vs C6706 | 10^7^ | 0.088873561 | 0.350328244 | 0.253686543 | 0.99985803 |
| E7946 vs AA142 | 10^7^ | 0.069742075 | 0.353625347 | 0.197220237 | 0.999959113 |
| AA142 vs HC1037 | 10^7^ | 0.059908321 | 0.352871741 | 0.169773644 | 0.999980564 |
| HC16 vs N16961 | 10^7^ | -0.039299824 | 0.35126096 | -0.11188213 | 0.999997562 |
| HC1037 vs HC16 | 10^8^ | -0.644999965 | 0.412957036 | -1.561905738 | 0.623817598 |
| E7946 vs HC1037 | 10^8^ | 0.59494063 | 0.409452338 | 1.45301559 | 0.694292185 |
| HC16 vs N16961 | 10^8^ | 0.517984496 | 0.417575827 | 1.240456134 | 0.816927179 |
| HC16 vs C6706 | 10^8^ | 0.486337103 | 0.418579367 | 1.161875481 | 0.854950407 |
| E7946 vs N16961 | 10^8^ | 0.467925162 | 0.414110313 | 1.129952932 | 0.869060926 |
| E7946 vs C6706 | 10^8^ | 0.436277769 | 0.415137717 | 1.050922985 | 0.900509578 |
| AA142 vs HC16 | 10^8^ | -0.415114018 | 0.422999465 | -0.981358258 | 0.924013994 |
| E7946 vs AA142 | 10^8^ | 0.365054684 | 0.419521249 | 0.870169709 | 0.953620465 |
| AA142 vs HC1037 | 10^8^ | 0.229885947 | 0.38881057 | 0.591254366 | 0.991648762 |
| HC1037 vs C6706 | 10^8^ | -0.158662862 | 0.383789148 | -0.413411537 | 0.998461411 |
| HC1037 vs N16961 | 10^8^ | -0.127015468 | 0.382756601 | -0.331843966 | 0.999469845 |
| AA142 vs N16961 | 10^8^ | 0.102870478 | 0.393715087 | 0.261281525 | 0.999835823 |
| AA142 vs C6706 | 10^8^ | 0.071223085 | 0.394791121 | 0.180407008 | 0.99997372 |
| E7946 vs HC16 | 10^8^ | -0.050059334 | 0.442038254 | -0.113246611 | 0.99999741 |
| C6706 vs N16961 | 10^8^ | 0.031647393 | 0.388785762 | 0.081400597 | 0.999999501 |
| AA142 vs HC16 | 10^9^ | -1.968940652 | 1.140103882 | -1.726983553 | 0.513751384 |
| E7946 vs HC16 | 10^9^ | -1.947134901 | 1.143440947 | -1.702873163 | 0.529805584 |
| AA142 vs C6706 | 10^9^ | -1.371132979 | 0.924880344 | -1.482497696 | 0.675557791 |
| E7946 vs C6706 | 10^9^ | -1.349327228 | 0.928682166 | -1.452948357 | 0.694334547 |
| HC1037 vs HC16 | 10^9^ | -1.684975434 | 1.164950828 | -1.446391894 | 0.698457273 |
| AA142 vs N16961 | 10^9^ | -1.236353936 | 0.870695282 | -1.41996168 | 0.714900125 |
| E7946 vs N16961 | 10^9^ | -1.214548185 | 0.874906863 | -1.388202831 | 0.734248845 |
| HC1037 vs C6706 | 10^9^ | -1.087167761 | 0.954932119 | -1.138476483 | 0.865371263 |
| HC1037 vs N16961 | 10^9^ | -0.952388718 | 0.902775303 | -1.054956548 | 0.899026477 |
| HC16 vs N16961 | 10^9^ | 0.732586716 | 1.294394993 | 0.565968441 | 0.993180758 |
| HC16 vs C6706 | 10^9^ | 0.597807673 | 1.331591531 | 0.448942231 | 0.997715113 |
| AA142 vs HC1037 | 10^9^ | -0.283965218 | 0.662781638 | -0.428444607 | 0.998173301 |
| E7946 vs HC1037 | 10^9^ | -0.262159467 | 0.668019917 | -0.392442591 | 0.998802925 |
| C6706 vs N16961 | 10^9^ | 0.134779043 | 1.109557547 | 0.12147098 | 0.999996327 |
| E7946 vs AA142 | 10^9^ | 0.021805751 | 0.624305916 | 0.034927991 | 0.999999993 |

p value adjustment method: Benjamini-Hochberg

Table S5. ANOVA of Larval CFU Model

| Term | Sum Sq | df | F Statistics | p Value |
| --- | --- | --- | --- | --- |
| Strain | 18.76910853 | 5 | 7.343638923 | < 0.001 |
| Time | 9.316820064 | 1 | 18.22658821 | < 0.001 |
| Cell Density | 342.7986176 | 3 | 223.5401207 | < 0.001 |
| Strain : Time | 11.50886143 | 5 | 4.502980131 | < 0.001 |
| Strain : Cell Density | 45.59001458 | 15 | 5.94588008 | < 0.001 |
| Time : Cell Density | 3.848084714 | 3 | 2.509348862 | 0.058426271 |
| Strain : Cell Density: Time | 33.34904559 | 15 | 4.349404747 | < 0.001 |
| Residuals | 201.3995742 | 394 | NA | NA |

Model Formula: Log_10_(CFU + 1) ~ Strain + Time + Cell Density + Strain : Time + Strain : Cell Density + Time : Cell Density + Strain : Cell Density: Time

Table S6. Statistic of Temporal Dynamic of Larval CFU

| Strain | Cell Density (cell/ml) | Time Trend (β) | Standard Error | df | t Statistics | p Value |
| --- | --- | --- | --- | --- | --- | --- |
| E7946 | 10^6^ | 0.002740647 | 0.009206284 | 394 | 0.297693036 | 0.766094364 |
| AA142 | 10^6^ | -0.023490206 | 0.00868037 | 394 | -2.706129506 | 0.007103047 |
| HC1037 | 10^6^ | -0.0057348 | 0.008409072 | 394 | -0.681977745 | 0.495653724 |
| HC16 | 10^6^ | -0.007697757 | 0.008672193 | 394 | -0.887636806 | 0.375277675 |
| C6706 | 10^6^ | 0.008643601 | 0.009006124 | 394 | 0.959747122 | 0.337771085 |
| N16961 | 10^6^ | 0.002907729 | 0.009009353 | 394 | 0.322745596 | 0.74705912 |
| E7946 | 10^7^ | -0.005635987 | 0.008279025 | 394 | -0.68075492 | 0.49642644 |
| AA142 | 10^7^ | -0.00222935 | 0.00827996 | 394 | -0.269246516 | 0.787880929 |
| HC1037 | 10^7^ | -0.004214191 | 0.009540423 | 394 | -0.441719483 | 0.65893454 |
| HC16 | 10^7^ | 0.005487665 | 0.007961702 | 394 | 0.689257819 | 0.491066743 |
| C6706 | 10^7^ | -0.008700033 | 0.009533236 | 394 | -0.912600234 | 0.362010893 |
| N16961 | 10^7^ | -0.014137917 | 0.009969628 | 394 | -1.418098814 | 0.156952339 |
| E7946 | 10^8^ | 0.00114553 | 0.007233642 | 394 | 0.158361433 | 0.874253137 |
| AA142 | 10^8^ | -0.016605503 | 0.008199417 | 394 | -2.025205375 | 0.043520052 |
| HC1037 | 10^8^ | -0.002100343 | 0.008672193 | 394 | -0.242192824 | 0.808756769 |
| HC16 | 10^8^ | 0.009466221 | 0.008199417 | 394 | 1.15449932 | 0.248995268 |
| C6706 | 10^8^ | -0.010263676 | 0.011340844 | 394 | -0.905018684 | 0.366008553 |
| N16961 | 10^8^ | -0.028634982 | 0.00996697 | 394 | -2.872987664 | 0.004286108 |
| E7946 | 10^9^ | -0.010671004 | 0.006840496 | 394 | -1.559975202 | 0.119568581 |
| AA142 | 10^9^ | -0.02059351 | 0.008279025 | 394 | -2.487431941 | 0.0132797 |
| HC1037 | 10^9^ | 0.019955288 | 0.00744749 | 394 | 2.679464885 | 0.007683094 |
| HC16 | 10^9^ | -0.05458694 | 0.007049591 | 394 | -7.743277279 | 8.26E-14 |
| C6706 | 10^9^ | 0.014868592 | 0.009972697 | 394 | 1.490929968 | 0.136780027 |
| N16961 | 10^9^ | -0.030077 | 0.0121617 | 394 | -2.473091722 | 0.013815955 |

p value adjustment method: Benjamini-Hochberg

Table S5. ANOVA of Water CFU Model

| Term | Sum Sq | df | F Statistics | p Value |
| --- | --- | --- | --- | --- |
| Strain | 34.8026183 | 5 | 3.696394829 | 0.002619705 |
| Cell Density | 970.1068587 | 3 | 171.7254885 | < 0.001 |
| Time | 370.5141131 | 1 | 196.761985 | < 0.001 |
| Larval Presence | 33.24940165 | 1 | 17.65713649 | < 0.001 |
| Strain : Cell Density | 92.51806862 | 15 | 3.275455767 | < 0.001 |
| Strain : Larval Presence | 3.235185463 | 5 | 0.34360986 | 0.886413828 |
| Cell Density : Time | 51.50550606 | 3 | 9.117354555 | < 0.001 |
| Cell Density : Larval Presence | 16.36454379 | 3 | 2.896803842 | 0.034452213 |
| Time : Larval Presence | 26.24367638 | 1 | 13.93673729 | < 0.001 |
| Residuals | 1284.245151 | 682 | NA | NA |

Model Formula: Log_10_(CFU + 1) ~ Strain + Cell Density + Time + Larval Presence + Strain : Cell Density + Strain : Larval Presence + Cell Density : Time + Cell Density : Larval Presence + Time : Larval Presence

Table S8. Pairwise Comparison of Water CFU

| Cell Density (cell/ml) | Strain | Larval Presence | Larval Presence | Estimate | df | Statistic | p Value |
| --- | --- | --- | --- | --- | --- | --- | --- |
| 10^6^ | AA142 | FALSE | TRUE | 0.301884201 | 682 | 0.983838023 | 0.325544051 |
| 10^6^ | C6706 | FALSE | TRUE | 0.650686804 | 682 | 2.120582715 | 0.034317256 |
| 10^6^ | E7946 | FALSE | TRUE | 0.441858162 | 682 | 1.440011963 | 0.15032288 |
| 10^6^ | HC1037 | FALSE | TRUE | 0.240189983 | 682 | 0.782777095 | 0.434029734 |
| 10^6^ | HC16 | FALSE | TRUE | 0.45690315 | 682 | 1.489043448 | 0.136938189 |
| 10^6^ | N16961 | FALSE | TRUE | 0.506723116 | 682 | 1.651406289 | 0.099115981 |
| 10^7^ | AA142 | FALSE | TRUE | 0.69549259 | 682 | 2.266604387 | 0.023726797 |
| 10^7^ | C6706 | FALSE | TRUE | 1.044295193 | 682 | 3.403349079 | < 0.001 |
| 10^7^ | E7946 | FALSE | TRUE | 0.835466551 | 682 | 2.722778327 | 0.006639034 |
| 10^7^ | HC1037 | FALSE | TRUE | 0.633798372 | 682 | 2.065543459 | 0.039248836 |
| 10^7^ | HC16 | FALSE | TRUE | 0.850511539 | 682 | 2.771809812 | 0.005726598 |
| 10^7^ | N16961 | FALSE | TRUE | 0.900331504 | 682 | 2.934172653 | 0.003456708 |
| 10^8^ | AA142 | FALSE | TRUE | 0.349678068 | 682 | 1.139597826 | 0.254853975 |
| 10^8^ | C6706 | FALSE | TRUE | 0.698480671 | 682 | 2.276342519 | 0.023134618 |
| 10^8^ | E7946 | FALSE | TRUE | 0.48965203 | 682 | 1.595771766 | 0.111003009 |
| 10^8^ | HC1037 | FALSE | TRUE | 0.28798385 | 682 | 0.938536898 | 0.348300821 |
| 10^8^ | HC16 | FALSE | TRUE | 0.504697017 | 682 | 1.644803252 | 0.100471196 |
| 10^8^ | N16961 | FALSE | TRUE | 0.554516983 | 682 | 1.807166092 | 0.071176935 |
| 10^9^ | AA142 | FALSE | TRUE | -0.152524207 | 682 | -0.497075082 | 0.619296305 |
| 10^9^ | C6706 | FALSE | TRUE | 0.196278396 | 682 | 0.63966961 | 0.522602225 |
| 10^9^ | E7946 | FALSE | TRUE | -0.012550245 | 682 | -0.040901142 | 0.967386677 |
| 10^9^ | HC1037 | FALSE | TRUE | -0.214218425 | 682 | -0.69813601 | 0.485330101 |
| 10^9^ | HC16 | FALSE | TRUE | 0.002494742 | 682 | 0.008130343 | 0.993515374 |
| 10^9^ | N16961 | FALSE | TRUE | 0.052314708 | 682 | 0.170493184 | 0.86467286 |

p value adjustment method: Benjamini-Hochberg

Table S9 Pairwise Comparison of Alpha Diversity Indices between Treated Samples and Control Samples

| Cell Density (cell/ml) | Time (h) | Contrast | Estimate | df | t Statistics | p Value | Index |
| --- | --- | --- | --- | --- | --- | --- | --- |
| 10^7^ | 24 | Control vs HC16 | 0.029684 | 139 | 1.041544 | 0.999958 | Simpson |
| 10^7^ | 24 | Control vs E7946 | 0.063617 | 139 | 2.145923 | 0.826656 | Simpson |
| 10^7^ | 24 | Control vs N16961 | 0.120119 | 139 | 4.051878 | 0.011957 | Simpson |
| 10^7^ | 24 | Control vs C6706 | 0.080135 | 139 | 2.788059 | 0.37554 | Simpson |
| 10^7^ | 48 | Control vs HC16 | 0.029684 | 139 | 1.041544 | 0.999958 | Simpson |
| 10^7^ | 48 | Control vs E7946 | 0.063617 | 139 | 2.145923 | 0.826656 | Simpson |
| 10^7^ | 48 | Control vs N16961 | 0.120119 | 139 | 4.051878 | 0.011957 | Simpson |
| 10^7^ | 48 | Control vs C6706 | 0.080135 | 139 | 2.788059 | 0.37554 | Simpson |
| 10^9^ | 24 | Control vs HC16 | 0.029684 | 139 | 1.041544 | 0.999958 | Simpson |
| 10^9^ | 24 | Control vs E7946 | 0.063617 | 139 | 2.145923 | 0.826656 | Simpson |
| 10^9^ | 24 | Control vs N16961 | 0.120119 | 139 | 4.051878 | 0.011957 | Simpson |
| 10^9^ | 24 | Control vs C6706 | 0.080135 | 139 | 2.788059 | 0.37554 | Simpson |
| 10^9^ | 48 | Control vs HC16 | 0.029684 | 139 | 1.041544 | 0.999958 | Simpson |
| 10^9^ | 48 | Control vs E7946 | 0.063617 | 139 | 2.145923 | 0.826656 | Simpson |
| 10^9^ | 48 | Control vs N16961 | 0.120119 | 139 | 4.051878 | 0.011957 | Simpson |
| 10^9^ | 48 | Control vs C6706 | 0.080135 | 139 | 2.788059 | 0.37554 | Simpson |
| 10^7^ | 24 | Control vs HC16 | 0.063381 | 140 | 0.7186 | 1 | Shannon |
| 10^7^ | 24 | Control vs E7946 | 0.173706 | 140 | 1.894817 | 0.934339 | Shannon |
| 10^7^ | 24 | Control vs N16961 | 0.274611 | 140 | 2.99551 | 0.249245 | Shannon |
| 10^7^ | 24 | Control vs C6706 | 0.192019 | 140 | 2.158608 | 0.819588 | Shannon |
| 10^7^ | 48 | Control vs HC16 | 0.063381 | 140 | 0.7186 | 1 | Shannon |
| 10^7^ | 48 | Control vs E7946 | 0.173706 | 140 | 1.894817 | 0.934339 | Shannon |
| 10^7^ | 48 | Control vs N16961 | 0.274611 | 140 | 2.99551 | 0.249245 | Shannon |
| 10^7^ | 48 | Control vs C6706 | 0.192019 | 140 | 2.158608 | 0.819588 | Shannon |
| 10^9^ | 24 | Control vs HC16 | 0.063381 | 140 | 0.7186 | 1 | Shannon |
| 10^9^ | 24 | Control vs E7946 | 0.173706 | 140 | 1.894817 | 0.934339 | Shannon |
| 10^9^ | 24 | Control vs N16961 | 0.274611 | 140 | 2.99551 | 0.249245 | Shannon |
| 10^9^ | 24 | Control vs C6706 | 0.192019 | 140 | 2.158608 | 0.819588 | Shannon |
| 10^9^ | 48 | Control vs HC16 | 0.063381 | 140 | 0.7186 | 1 | Shannon |
| 10^9^ | 48 | Control vs E7946 | 0.173706 | 140 | 1.894817 | 0.934339 | Shannon |
| 10^9^ | 48 | Control vs N16961 | 0.274611 | 140 | 2.99551 | 0.249245 | Shannon |
| 10^9^ | 48 | Control vs C6706 | 0.192019 | 140 | 2.158608 | 0.819588 | Shannon |
| 10^7^ | 24 | Control vs HC16 | -1.39747 | 140 | -0.84081 | 0.999999 | PD |
| 10^7^ | 24 | Control vs E7946 | -0.23131 | 140 | -0.1339 | 1 | PD |
| 10^7^ | 24 | Control vs N16961 | -0.5433 | 140 | -0.3145 | 1 | PD |
| 10^7^ | 24 | Control vs C6706 | -2.00463 | 140 | -1.19588 | 0.999687 | PD |
| 10^7^ | 48 | Control vs HC16 | -1.39747 | 140 | -0.84081 | 0.999999 | PD |
| 10^7^ | 48 | Control vs E7946 | -0.23131 | 140 | -0.1339 | 1 | PD |
| 10^7^ | 48 | Control vs N16961 | -0.5433 | 140 | -0.3145 | 1 | PD |
| 10^7^ | 48 | Control vs C6706 | -2.00463 | 140 | -1.19588 | 0.999687 | PD |
| 10^9^ | 24 | Control vs HC16 | -1.39747 | 140 | -0.84081 | 0.999999 | PD |
| 10^9^ | 24 | Control vs E7946 | -0.23131 | 140 | -0.1339 | 1 | PD |
| 10^9^ | 24 | Control vs N16961 | -0.5433 | 140 | -0.3145 | 1 | PD |
| 10^9^ | 24 | Control vs C6706 | -2.00463 | 140 | -1.19588 | 0.999687 | PD |
| 10^9^ | 48 | Control vs HC16 | -1.39747 | 140 | -0.84081 | 0.999999 | PD |
| 10^9^ | 48 | Control vs E7946 | -0.23131 | 140 | -0.1339 | 1 | PD |
| 10^9^ | 48 | Control vs N16961 | -0.5433 | 140 | -0.3145 | 1 | PD |
| 10^9^ | 48 | Control vs C6706 | -2.00463 | 140 | -1.19588 | 0.999687 | PD |
| 10^7^ | 24 | Control vs HC16 | -28.7614 | 140 | -1.55013 | 0.99134 | Chao1 |
| 10^7^ | 24 | Control vs E7946 | -7.42635 | 140 | -0.38509 | 1 | Chao1 |
| 10^7^ | 24 | Control vs N16961 | -11.9295 | 140 | -0.61859 | 1 | Chao1 |
| 10^7^ | 24 | Control vs C6706 | -21.3345 | 140 | -1.1401 | 0.999841 | Chao1 |
| 10^7^ | 48 | Control vs HC16 | -28.7614 | 140 | -1.55013 | 0.99134 | Chao1 |
| 10^7^ | 48 | Control vs E7946 | -7.42635 | 140 | -0.38509 | 1 | Chao1 |
| 10^7^ | 48 | Control vs N16961 | -11.9295 | 140 | -0.61859 | 1 | Chao1 |
| 10^7^ | 48 | Control vs C6706 | -21.3345 | 140 | -1.1401 | 0.999841 | Chao1 |
| 10^9^ | 24 | Control vs HC16 | -28.7614 | 140 | -1.55013 | 0.99134 | Chao1 |
| 10^9^ | 24 | Control vs E7946 | -7.42635 | 140 | -0.38509 | 1 | Chao1 |
| 10^9^ | 24 | Control vs N16961 | -11.9295 | 140 | -0.61859 | 1 | Chao1 |
| 10^9^ | 24 | Control vs C6706 | -21.3345 | 140 | -1.1401 | 0.999841 | Chao1 |
| 10^9^ | 48 | Control vs HC16 | -28.7614 | 140 | -1.55013 | 0.99134 | Chao1 |
| 10^9^ | 48 | Control vs E7946 | -7.42635 | 140 | -0.38509 | 1 | Chao1 |
| 10^9^ | 48 | Control vs N16961 | -11.9295 | 140 | -0.61859 | 1 | Chao1 |
| 10^9^ | 48 | Control vs C6706 | -21.3345 | 140 | -1.1401 | 0.999841 | Chao1 |
| 10^7^ | 24 | Control vs HC16 | -29.4056 | 140 | -1.59081 | 0.988416 | ACE |
| 10^7^ | 24 | Control vs E7946 | -7.23599 | 140 | -0.37663 | 1 | ACE |
| 10^7^ | 24 | Control vs N16961 | -12.0199 | 140 | -0.62563 | 1 | ACE |
| 10^7^ | 24 | Control vs C6706 | -21.643 | 140 | -1.16094 | 0.999794 | ACE |
| 10^7^ | 48 | Control vs HC16 | -29.4056 | 140 | -1.59081 | 0.988416 | ACE |
| 10^7^ | 48 | Control vs E7946 | -7.23599 | 140 | -0.37663 | 1 | ACE |
| 10^7^ | 48 | Control vs N16961 | -12.0199 | 140 | -0.62563 | 1 | ACE |
| 10^7^ | 48 | Control vs C6706 | -21.643 | 140 | -1.16094 | 0.999794 | ACE |
| 10^9^ | 24 | Control vs HC16 | -29.4056 | 140 | -1.59081 | 0.988416 | ACE |
| 10^9^ | 24 | Control vs E7946 | -7.23599 | 140 | -0.37663 | 1 | ACE |
| 10^9^ | 24 | Control vs N16961 | -12.0199 | 140 | -0.62563 | 1 | ACE |
| 10^9^ | 24 | Control vs C6706 | -21.643 | 140 | -1.16094 | 0.999794 | ACE |
| 10^9^ | 48 | Control vs HC16 | -29.4056 | 140 | -1.59081 | 0.988416 | ACE |
| 10^9^ | 48 | Control vs E7946 | -7.23599 | 140 | -0.37663 | 1 | ACE |
| 10^9^ | 48 | Control vs N16961 | -12.0199 | 140 | -0.62563 | 1 | ACE |
| 10^9^ | 48 | Control vs C6706 | -21.643 | 140 | -1.16094 | 0.999794 | ACE |
| 10^7^ | 24 | Control vs HC16 | -25.9671 | 140 | -1.52961 | 0.992566 | Observed |
| 10^7^ | 24 | Control vs E7946 | -6.80109 | 140 | -0.38544 | 1 | Observed |
| 10^7^ | 24 | Control vs N16961 | -9.68998 | 140 | -0.54917 | 1 | Observed |
| 10^7^ | 24 | Control vs C6706 | -19.0814 | 140 | -1.11448 | 0.999886 | Observed |
| 10^7^ | 48 | Control vs HC16 | -25.9671 | 140 | -1.52961 | 0.992566 | Observed |
| 10^7^ | 48 | Control vs E7946 | -6.80109 | 140 | -0.38544 | 1 | Observed |
| 10^7^ | 48 | Control vs N16961 | -9.68998 | 140 | -0.54917 | 1 | Observed |
| 10^7^ | 48 | Control vs C6706 | -19.0814 | 140 | -1.11448 | 0.999886 | Observed |
| 10^9^ | 24 | Control vs HC16 | -25.9671 | 140 | -1.52961 | 0.992566 | Observed |
| 10^9^ | 24 | Control vs E7946 | -6.80109 | 140 | -0.38544 | 1 | Observed |
| 10^9^ | 24 | Control vs N16961 | -9.68998 | 140 | -0.54917 | 1 | Observed |
| 10^9^ | 24 | Control vs C6706 | -19.0814 | 140 | -1.11448 | 0.999886 | Observed |
| 10^9^ | 48 | Control vs HC16 | -25.9671 | 140 | -1.52961 | 0.992566 | Observed |
| 10^9^ | 48 | Control vs E7946 | -6.80109 | 140 | -0.38544 | 1 | Observed |
| 10^9^ | 48 | Control vs N16961 | -9.68998 | 140 | -0.54917 | 1 | Observed |
| 10^9^ | 48 | Control vs C6706 | -19.0814 | 140 | -1.11448 | 0.999886 | Observed |

p value adjustment method: Benjamini-Hochberg
